# Melatonin Attenuates Cadmium-Induced Oxidative Stress in Potato by Remodeling Redox Homeostasis and Activating Multi-Pathway Antioxidant Networks

**DOI:** 10.3390/antiox15091180

**Published:** 2026-09-17

**Authors:** Panfeng Yao, Junmei Cui, Wenlin Li, Jianguo Wei, Ying Wang, Chao Sun, Zhenzhen Bi, Zhen Liu, Han Wang, Yuhui Liu, Jiangping Bai

**Affiliations:** 1 State Key Laboratory of Aridland Crop Science, Gansu Agricultural University, Lanzhou 730070, China; yaopf@gsau.edu.cn (P.Y.); cuijm@gsau.edu.cn (J.C.); 1073325121094@st.gsau.edu.cn (J.W.); 1073325121103@st.gsau.edu.cn (Y.W.); sunc@gsau.edu.cn (C.S.); bizz@gsau.edu.cn (Z.B.); liuzhen@gsau.edu.cn (Z.L.); 2 College of Agronomy/Seed Industry Research Institute of Gansu Provincial University, Gansu Agricultural University, Lanzhou 730070, China; wh@gsau.edu.cn; 3 College of Horticulture, Gansu Agricultural University, Lanzhou 730070, China; liwenl@gsau.edu.cn; 4 School of Materials Science and Engineering, Lanzhou Institute of Technology, Lanzhou 730070, China

**Keywords:** potato, cadmium stress, melatonin, transcriptomics, antioxidant defense

## Abstract

Cadmium pollution severely suppresses potato growth, impairs photosynthesis, and disrupts cellular redox balance, threatening crop yield and food safety. Exogenous small-molecule regulators represent low-cost strategies to alleviate heavy-metal phytotoxicity, yet systematic comparisons of N-acetyl-L-cysteine (NAC), L-tryptophan (Trp), and melatonin (MT) on Cd-stressed potatoes remain insufficient. In this study, two rounds of concentration-gradient screening using morphological and physiological assays identified 5 μM of melatonin as the optimal treatment to alleviate cadmium-induced damage in potato seedlings. This concentration significantly restored shoot height, root length, fresh weight, and chlorophyll content. Relative to the Cd-only control, 5 μM of MT reduced MDA and cadmium accumulation by 51.88% and 49.26%, respectively, while increasing proline content by 40.99%. Transcriptomic analysis revealed that MT reversed Cd-induced suppression of photosynthesis and carbon fixation pathways and activated carbohydrate metabolism. Weighted gene co-expression network analysis identified four key modules significantly correlated with Cd tolerance and MT-mediated recovery. Eight hub genes, including *StANNAT1* (Ca^2+^-ROS homeostasis), *StNRT1.1* (nitrogen transport), *StSWEET10* (carbon allocation), and *StHMG1* (MVA pathway), were validated by qPCR, confirming their involvement in Ca^2+^-ROS signaling, membrane integrity, and photosynthetic recovery. Mechanistically, MT rebalanced cellular redox status, promoted Cd sequestration, optimized carbon–nitrogen distribution, and enhanced antioxidant defense. These findings demonstrate that exogenous MT alleviates Cd toxicity through coordinated regulation of multiple pathways, providing a practical strategy for safe potato cultivation in Cd-contaminated soils and establishing a molecular framework for understanding MT-mediated heavy-metal tolerance in crops.

## 1. Introduction

Cadmium (Cd) is a highly mobile and phytotoxic heavy-metal pollutant widely accumulated in agricultural soil. Excessive Cd absorption severely impairs crop growth and metabolism at multiple levels [1]. Phenotypically, Cd stress restricts root development and reduces plant biomass. Physiologically, it triggers massive reactive oxygen species bursts, disrupts cellular redox homeostasis, and damages photosynthetic systems. In terms of agricultural production, Cd toxicity declines crop yield and deteriorates nutritional and commercial quality of edible organs. As a non-degradable toxic element, Cd can be continuously enriched in crops and enter the food chain, posing serious hidden dangers to food safety and human health [2].

Potato (*Solanum tuberosum* L.) is the fourth most important food crop worldwide after rice, wheat, and maize. It plays a crucial role in ensuring global food security and economic-crop industrial development. In recent decades, rapid industrialization, sewage irrigation, and excessive application of chemical fertilizers have caused continuous Cd accumulation in farmland soil [3,4]. A large number of agricultural soils have suffered varying degrees of Cd contamination. Previous field investigations have confirmed that Cd pollution significantly suppresses potato growth and tuber yield [5,6]. Mild and moderate Cd stress can reduce potato single-plant yield and dry weight by more than 30% [7]. Severe Cd pollution further decreases tuber starch content and dry matter accumulation rate, which directly deteriorates potato commercial quality. In addition, potato tubers show strong Cd enrichment capacity. The Cd concentration in edible tubers in polluted farmland often exceeds the national food safety limit [6,8]. This phenomenon severely restricts the sustainable development of the potato industry and causes potential food safety risks.

To alleviate Cd phytotoxicity and ensure safe crop production in contaminated farmland, multiple mitigation strategies have been widely explored in recent years. Conventional approaches include tolerant cultivar screening, stress-resistant gene excavation, and transgenic breeding [9,10]. However, these methods require long breeding cycles and face limitations in field popularization and application. In contrast, exogenous application of small-molecule bioactive regulators has become a rapid, efficient, and low-cost mitigation strategy for heavy-metal stress [11]. This technique can effectively improve plant antioxidant capacity and heavy-metal detoxification ability. A variety of exogenous substances have been verified to alleviate Cd toxicity in crops. For example, exogenous salicylic acid can enhance antioxidant enzyme activity and reduce ROS accumulation under Cd stress [12,13]. Exogenous proline and glycine betaine can stabilize cell membrane structure and alleviate Cd-induced osmotic stress [14]. Exogenous silicon and zinc can block Cd absorption and transport by regulating metal ion transporters and reduce Cd accumulation in edible tissues.

Melatonin (MT) and L-tryptophan (L-Trp) are two classic plant endogenous regulatory molecules with excellent antioxidant and stress-resistant functions [15]. L-Trp acts as a key precursor substance for melatonin biosynthesis in plants. Exogenous L-Trp supplementation effectively increases endogenous melatonin accumulation under abiotic stress [16]. Melatonin serves as a multi-functional antioxidant and signal molecule. It directly scavenges excess ROS and enhances the activity of antioxidant enzymes, including SOD, POD, and CAT, under Cd stress. It also regulates the expression of heavy-metal transport and compartmentalization related genes. This process reduces Cd absorption in roots and promotes Cd isolation in vacuoles, thereby reducing Cd accumulation in aboveground tissues and edible organs. N-acetyl-L-cysteine (NAC) is an important precursor of glutathione synthesis. It participates in plant antioxidant defense and heavy-metal chelation metabolism [17,18]. Although the regulatory effects of these three regulators have been reported in various plant species, systematic comparative studies on their Cd-alleviation efficiency in potato are still insufficient. Most previous studies adopted single-concentration treatment and lacked gradient-optimization verification. The optimal application dosage and core transcriptional regulatory pathways of MT, L-Trp, and NAC in mitigating potato Cd stress remain unclear.

In this study, three exogenous regulators, namely NAC, L-Trp, and MT, were selected for two rounds of gradient concentration screening. A series of morphological and physiological indexes were determined to compare their Cd-alleviation effects on potato plantlets. The optimal melatonin concentration with the best mitigation effect was finally screened out. Transcriptome sequencing was performed on four representative treatment groups to explore key functional genes and metabolic pathways responding to melatonin-mediated Cd tolerance. WGCNA, combined with phenotypic physiological data, was used to mine core hub genes closely related to Cd detoxification and antioxidant regulation. qRT-PCR was conducted to verify the expression patterns of key candidate genes. This study aims to clarify the molecular mechanism of exogenous melatonin alleviating cadmium toxicity in potato. It provides a theoretical basis and technical reference for the application of small-molecule regulators in safe potato production in Cd-contaminated farmland.

## 2. Materials and Methods

### 2.1. Plant Materials and Culture Conditions

The commercial potato cultivar “Atlantic”, a widely planted processing variety, was used in this study. Sterile tissue-cultured plantlets were continuously preserved and subcultured in our laboratory. Uniform plantlets were maintained on solid Murashige and Skoog (MS) medium and subcultured every three weeks to ensure consistent growth status. The tissue culture environment was maintained at 22 ± 2 °C with a 16 h light/8 h dark photoperiod, and the light intensity was set at 30–35 μmol m^−2^ s^−1^. After consistent growth and vigorous root development, uniformly sized plantlets were selected for subsequent Cd stress and exogenous substance treatments.

### 2.2. Chemical Reagents and Treatment Concentration Design

Three exogenous regulators were applied in this study. The reagents included N-acetyl-L-cysteine (NAC, Cat. No. A7250, ≥99% purity, Sigma-Aldrich, St. Louis, MO, USA), L-tryptophan (L-Trp, Cat. No. 1.08374, Sigma-Aldrich), and melatonin (MT, Cat. No. 461326, ≥99.5% HPLC, Sigma-Aldrich). Cadmium stress was induced by anhydrous cadmium chloride (CdCl_2_, Cat. No. C2544, ACS reagent grade, Sigma-Aldrich). The fixed cadmium concentration used in all treatments was 100 μM.

Stage-one concentration screening was conducted for all three regulators. Each regulator contained eight experimental groups. The groups were control (CK), single cadmium treatment (Cd), three single regulator treatments, and three combined cadmium plus regulator treatments. Three initial concentration levels were set for each reagent. The NAC concentrations were 125 μM, 250 μM, and 500 μM (marked NAC1, NAC2, and NAC3). The L-Trp concentrations were 25 μM, 50 μM, and 100 μM (marked Trp1, Trp2, and Trp3). The MT concentrations were 25 μM, 50 μM, and 100 μM (marked MT1, MT2, and MT3).

Stage-two refined concentration screening was only performed on Trp and MT. Preliminary physiological data showed that NAC had no obvious alleviating effect on cadmium toxicity. Four refined concentrations were designed for Trp and MT. Each regulator contained ten experimental groups: control, single cadmium stress, four single regulator treatments, and four combined cadmium regulator treatments. The refined Trp concentrations were 5 μM, 10 μM, 15 μM, and 20 μM (marked Trp4, Trp5, Trp6, and Trp7). The refined MT concentrations were 5 μM, 10 μM, 15 μM, and 20 μM (marked MT4, MT5, MT6, and MT7).

Stock solutions of MT, Trp, and NAC were prepared at 100× final concentrations, filter-sterilized through 0.22 μm sterile filters, and stored at 4 °C in the dark until use. MS medium was autoclaved and then cooled to approximately 45–50 °C before the stock solutions were added aseptically. The final concentrations were calculated based on the added stock volumes. To minimize potential degradation, all stock solutions were prepared fresh before each experiment, kept protected from light, and added to the medium only after it had cooled to below 50 °C. The actual concentrations of MT, Trp, and NAC in the solidified medium were not analytically verified in this study; however, the same filter-sterilization and post-autoclaving supplementation procedure has been widely adopted in previous plant tissue-culture studies for these compounds, and the observed concentration-dependent physiological responses in our experiments indirectly support the stability and bioavailability of the supplemented compounds.

### 2.3. Morphological Trait Measurement

After 21 days of treatment, potato plantlets were harvested and rinsed with distilled water to remove residual medium. For aboveground morphological observation, plantlets were placed flat on a black background with a scale bar and photographed vertically using a digital camera. Leaf number was counted, and shoot fresh weight was determined using an electronic balance.

For root morphological analysis, root systems were carefully separated and thoroughly cleaned. Clean roots were evenly spread on the scanning tray without overlapping and scanned using an Epson Expression 12000XL flatbed scanner (Epson, Suwa, Nagano, Japan). Root morphological parameters, including total root surface area, average root diameter, total root volume, number of root branching, and the ratio of root length to root volume, were automatically calculated via WinRHIZO 5.0 software (Regent Instruments, Quebec City, QC, Canada). The experiment was performed with three independent biological replicates.

### 2.4. Physiological Biochemical Index Determination

Fully expanded functional leaves were harvested from plantlets after 21 days of treatment. Midribs were removed, and leaf tissues were cut into small pieces and mixed thoroughly.

Chlorophyll content was determined by acetone extraction [19]. Fresh leaf tissue (0.2 g) was placed in 10 mL centrifuge tubes with 10 mL of 80% acetone. Tubes were sealed and kept in darkness at 4 °C for approximately 48 h, with gentle shaking every 1 h until the tissue became completely white. After centrifugation at 5000× *g* for 10 min at 4 °C, the supernatant was collected. Absorbance was read at 663, 645, 470, and 652 nm using 80% acetone as blank. Chlorophyll content was calculated according to the following formula: chlorophyll content (%) = C × V/(1000 × W), where C is the chlorophyll concentration calculated from the absorbance values, V is the volume of 80% acetone (mL), and W is the fresh weight of the leaf tissue (g).

Malondialdehyde (MDA) content was determined using the thiobarbituric acid (TBA) method [20]. Fresh leaf tissue (0.5 g) was homogenized in 5 mL of 10% (*w*/*v*) trichloroacetic acid (TCA) and centrifuged at 10,000× *g* for 15 min. The supernatant (2 mL) was mixed with 2 mL of 0.6% (*w*/*v*) TBA in 10% TCA. The mixture was heated at 95 °C for 30 min, quickly cooled on ice, and centrifuged. Absorbance was read at 532, 600, and 450 nm. MDA content was calculated using the formula MDA (μM) = 6.45 × (A_532_ − A_600_) − 0.56 × A_450_, and expressed as μmol g^−1^ FW.

Proline content was quantified using the acid ninhydrin method [21]. Fresh leaf tissue (0.5 g) was homogenized in 5 mL of 3% sulfosalicylic acid and incubated in a boiling water bath for 10 min. After centrifugation, 2 mL of the supernatant was reacted with 2 mL of glacial acetic acid and 2 mL of acid ninhydrin reagent at 100 °C for 30 min. The reaction mixture was extracted with 4 mL of toluene, and the absorbance of the toluene phase was read at 520 nm. Proline concentration was determined from a standard curve and expressed as μmol g^−1^ FW.

### 2.5. Determination of Cadmium Concentration in Potato Seedlings

After harvesting, potato seedling samples were rinsed three times with de-ionized water to remove cadmium residue attached to the root surface. In this study, whole potato plantlets (combined roots and shoots) were used for Cd quantification. All plant tissues were oven-dried at 105 °C for 30 min, followed by drying to constant weight at 75 °C. The dried samples were ground into fine powder using a mill and passed through a 0.15 mm sieve. Approximately 0.1 g of plant powder was weighed and digested with a mixed acid solution (HNO_3_:HClO_4_, 4:1, *v*/*v*). The digestion was performed on a graphite digestion block using a stepwise temperature program: 120 °C for 1 h, and 160 °C for 2 h, until the digest became clear and nearly colorless. After complete digestion, the digested solution was diluted to a final volume of 25 mL with ultrapure water. The cadmium content was determined by inductively coupled plasma–optical emission spectrometry (ICP-OES, Agilent 5110, Agilent Technologies, Santa Clara, CA, USA). The instrument was operated with a radio frequency power of 1150 W, a nebulizer gas flow rate of 0.7 L/min, and an integration time of 5 s. Cadmium was quantified at an analytical wavelength of 228.8 nm. A calibration curve was prepared using a series of Cd standard solutions (0, 0.5, 1.0, 1.5, 2.0, and 3.0 μg/L) with a linear correlation coefficient (R^2^) greater than 0.999. Roots and shoots were harvested and analyzed as a combined whole-plantlet sample. The Cd concentration was calculated as follows: Cd content (mg/kg DW) = (ρ − ρ_0_) × V/(m × 1000), where ρ is the Cd concentration in the sample solution (μg/L), ρ_0_ is the Cd concentration in the blank (μg/L), V is the final volume (mL), and m is the sample weight (g). The results were expressed as mg/kg dry weight (DW). Each treatment included three independent biological replicates.

### 2.6. Transcriptome Sequencing Sample Collection

Based on comprehensive morphological and physiological data obtained from two rounds of concentration screening, 5 μM of melatonin was identified as the optimal treatment concentration. The four groups were CK, Cd, MT, and Cd + MT. Three biological replicates were collected for each group. Fresh leaf tissues were rapidly sampled and frozen in liquid nitrogen. All tissue samples were preserved at minus 80 °C before RNA extraction.

### 2.7. RNA Extraction and Transcriptome Sequencing

Total RNA was extracted from potato leaf tissues using an RNA-extraction kit. RNA purity and integrity were evaluated by agarose gel electrophoresis and micro-spectrophotometry. High-quality RNA was used to construct cDNA libraries for paired-end transcriptome sequencing on an Illumina platform (Illumina Inc., San Diego, CA, USA). Raw reads were filtered to remove adapter sequences and low-quality reads. Clean reads were mapped to the potato reference genome, and gene-expression levels were quantified as FPKM values.

### 2.8. Differential Expression Gene Identification and Functional Enrichment

Differentially expressed genes (DEGs) were screened with a threshold of |log2 fold change| ≥ 1 and adjusted *p*-value < 0.05. Venn analysis was conducted to analyze shared and specific DEGs among comparison groups. Gene ontology (GO) enrichment and Kyoto Encyclopedia of Genes and Genomes (KEGG) pathway enrichment were performed on all differential genes. Top enriched biological processes and metabolic pathways were visualized by bubble charts.

### 2.9. Weighted Gene Co-Expression Network Analysis (WGCNA)

WGCNA was performed using the normalized gene expression matrix. To reduce noise caused by limited sample size, genes with low expression variation were filtered prior to network construction. An unsigned co-expression network was built with an optimized soft threshold power to meet the approximate scale-free topology criterion. The minimum module size was set to 50 to exclude trivial small modules derived from random noise. Module preservation analysis with permutation tests was carried out to evaluate network robustness. Module eigengenes were correlated with all measured morphological and physiological traits. Genes satisfying both high module membership (MM > 0.8) and high gene significance (GS > 0.7) toward cadmium tolerance were regarded as candidate hub genes.

### 2.10. Quantitative Real-Time PCR Verification

Total RNA was isolated from liquid nitrogen-frozen potato plantlets under Cd treatment using the RN33 kit (Aidlab Biotech, Beijing, China). Purified RNA was reverse-transcribed into cDNA with ReverTra Ace^®^ qPCR RT Master Mix (TOYOBO, Osaka, Japan). RT-qPCR assays were conducted on a LightCycler^®^ 96 System (Roche, Basel, Switzerland) using LightCycler 480 SYBR Green I Master mix. The 10 μL reaction system contained 5 μL of 2 × SYBR Green master mix, 0.4 μL of primer mix, and 200 ng of cDNA template. Amplification procedures included an initial denaturation at 95 °C for 30 s, followed by 39 cycles of 98 °C for 5 s, 60 °C for 30 s, and a melting curve stage at 98 °C for 15 s. Relative gene expression was calculated by the 2^−ΔΔCt^ method [22]. All RT-qPCR primer sequences are provided in Supplementary Table S1.

### 2.11. Statistical Analysis

All statistical analyses were performed using SPSS 26.0 (IBM Corporation, Armonk, NY, USA). Independent-samples Student’s *t*-tests were conducted as planned pairwise comparisons, in which each regulator-treated group was compared only with its corresponding control: Trp groups without Cd were compared with the CK control, whereas Cd + Trp groups were compared with the Cd-only control. No correction for multiple comparisons was applied, as these were predefined, limited pairwise comparisons rather than an exhaustive test of all possible group combinations. Statistical significance was defined at * *p* < 0.05 and ** *p* < 0.01. Principal component analysis, cluster analysis, and correlation analysis were performed using R packages (v 4.2.1), and all figures were plotted in GraphPad Prism (version 11.1.0) (GraphPad Software, San Diego, CA, USA). In all figures, asterisks denote a significant difference relative to the corresponding control group as described above.

## 3. Results

### 3.1. Screening of Exogenous Regulators for Alleviating Cd Toxicity in Potato

**Figure 1:**
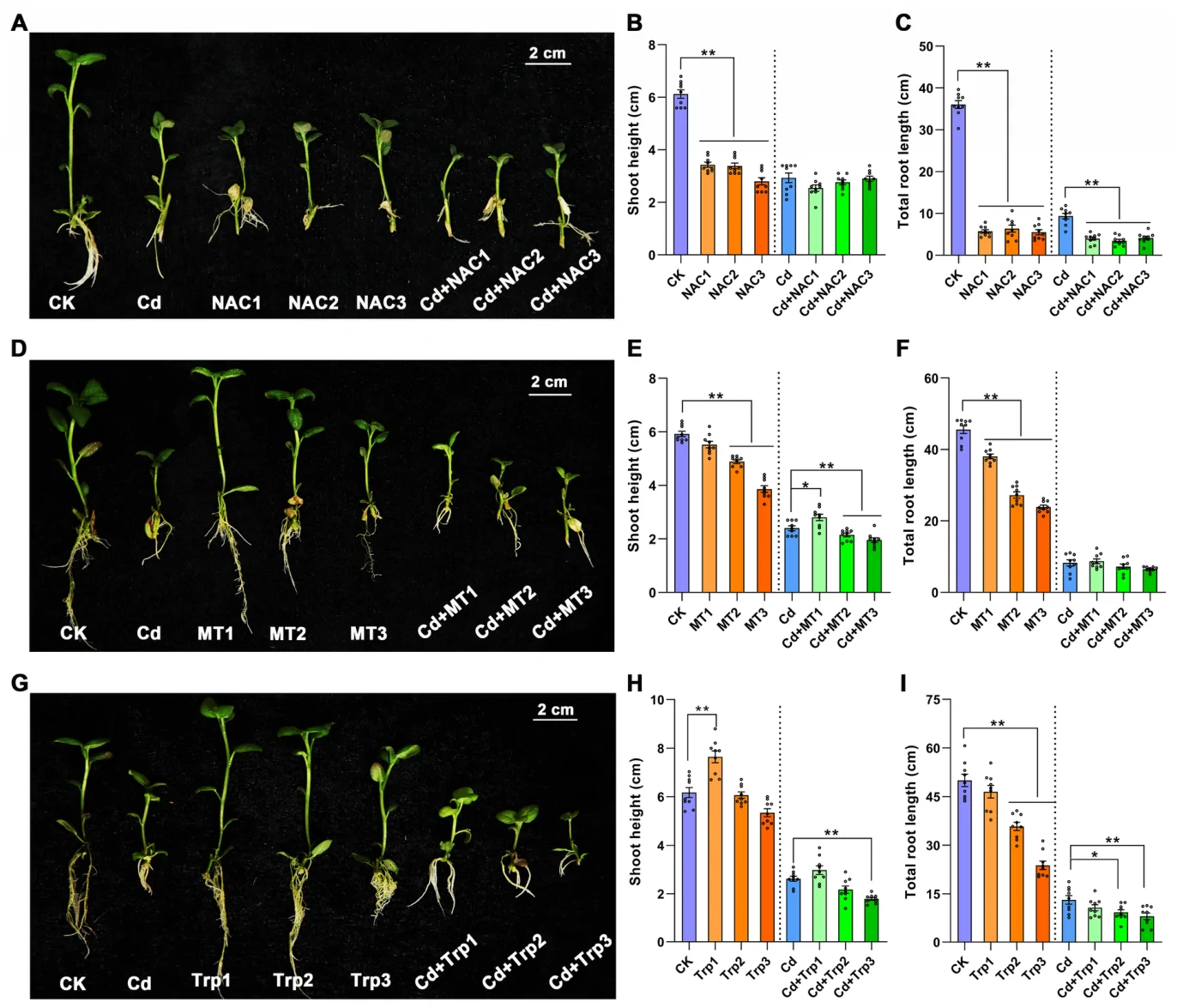
Phenotypic screening of potato plantlets under cadmium stress after treatment with three exogenous regulators at three concentration gradients. (**A**,**D**,**G**) Morphological photographs of potato plantlets under different treatments; scale bar = 2 cm. (**B**,**C**) Shoot height and total root length of plantlets with NAC application under normal and Cd stress conditions. (**E**,**F**) Shoot height and total root length of plantlets treated with melatonin. (**H**,**I**) Shoot height and total root length of plantlets treated with tryptophan. Planned pairwise comparisons were performed using independent-samples Student’s *t*-test. Asterisks denote significant differences relative to the corresponding control group (non-Cd treatments vs. CK; Cd-containing treatments vs. Cd-only control): * and ** indicate *p* < 0.05 and *p* < 0.01, respectively. Error bars represent standard error (SE) of biological replicates.

Cadmium stress severely inhibited the growth of potato plantlets. Compared with the CK group, Cd treatment reduced shoot height by approximately 56.31%, total root length by 76.37%, shoot fresh weight by 65.28%, and leaf number by 53.71% (Figure 1). Root architecture was also significantly damaged; total root surface area and root branching number declined by 65.94% and 78.94%, respectively.

Supplementation with NAC failed to relieve cadmium-induced growth restriction within the tested concentration range. All combined Cd + NAC groups exhibited comparable shoot height, root length, shoot fresh weight, and leaf number to the single Cd treatment (Figure 1A–C). In addition, NAC supplementation failed to restore root growth, and several root morphological parameters were even lower than those observed under cadmium treatment alone in this study (Figures S1A and S2A–E). These findings suggest that NAC, at least within the tested concentration range and experimental conditions, exerted no obvious alleviating effect on Cd toxicity in potato plantlets. Further studies with lower concentration gradients and direct measurements of GSH metabolism may be needed to fully evaluate the potential of NAC.

**Figure 2:**
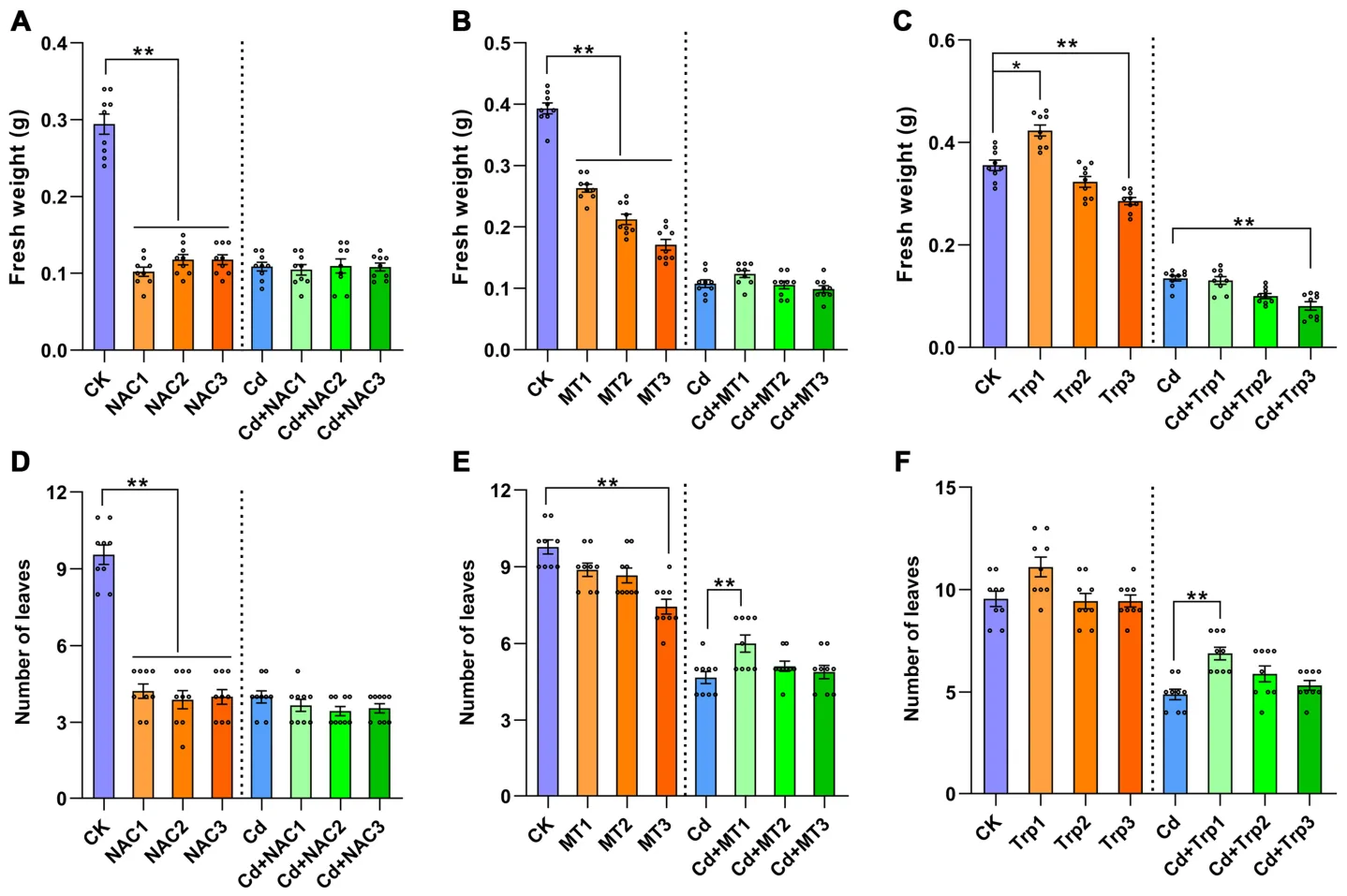
Fresh weight and leaf number of potato plantlets under Cd stress with three exogenous regulators at three concentrations. (**A**,**D**) NAC treatments, (**B**,**E**) MT treatments, and (**C**,**F**) Trp treatments. Planned pairwise comparisons were performed using independent-samples Student’s *t*-test. Asterisks denote significant differences relative to the corresponding control group (non-Cd treatments vs. CK; Cd-containing treatments vs. Cd-only control): * and ** indicate *p* < 0.05 and *p* < 0.01, respectively. Error bars represent standard error (SE) of biological replicates.

Trp and melatonin exerted distinct protective effects against cadmium stress, with their alleviating performances showing obvious concentration dependence (Figures S1B,C, S3 and S4). The lowest tested concentration (25 μM) of both substances generated markedly stronger protective effects relative to 50 μM and 100 μM treatments. Compared with the sole Cd group, the Cd + MT1 treatment raised shoot height by 16.80% and leaf number by 23.81%, while Cd + Trp1 increased leaf number by 40.91%. In contrast, the highest-concentration treatments (Cd + MT3 and Cd + Trp3) only slightly alleviated cadmium-induced growth damage, and even inhibited several growth traits to a certain degree (Figure 1D–I and Figure 2A–F).

The observation that 25 μM generated stronger effects than higher concentrations implied that optimal mitigation performance might exist at concentrations below 25 μM. Accordingly, refined screening with lower concentration gradients was conducted for Trp and MT in subsequent experiments.

### 3.2. Refined Screening of Low-Concentration Trp for Cd Stress Alleviation

#### 3.2.1. Normal Growth Performance Under Single Trp Treatment

**Figure 3:**
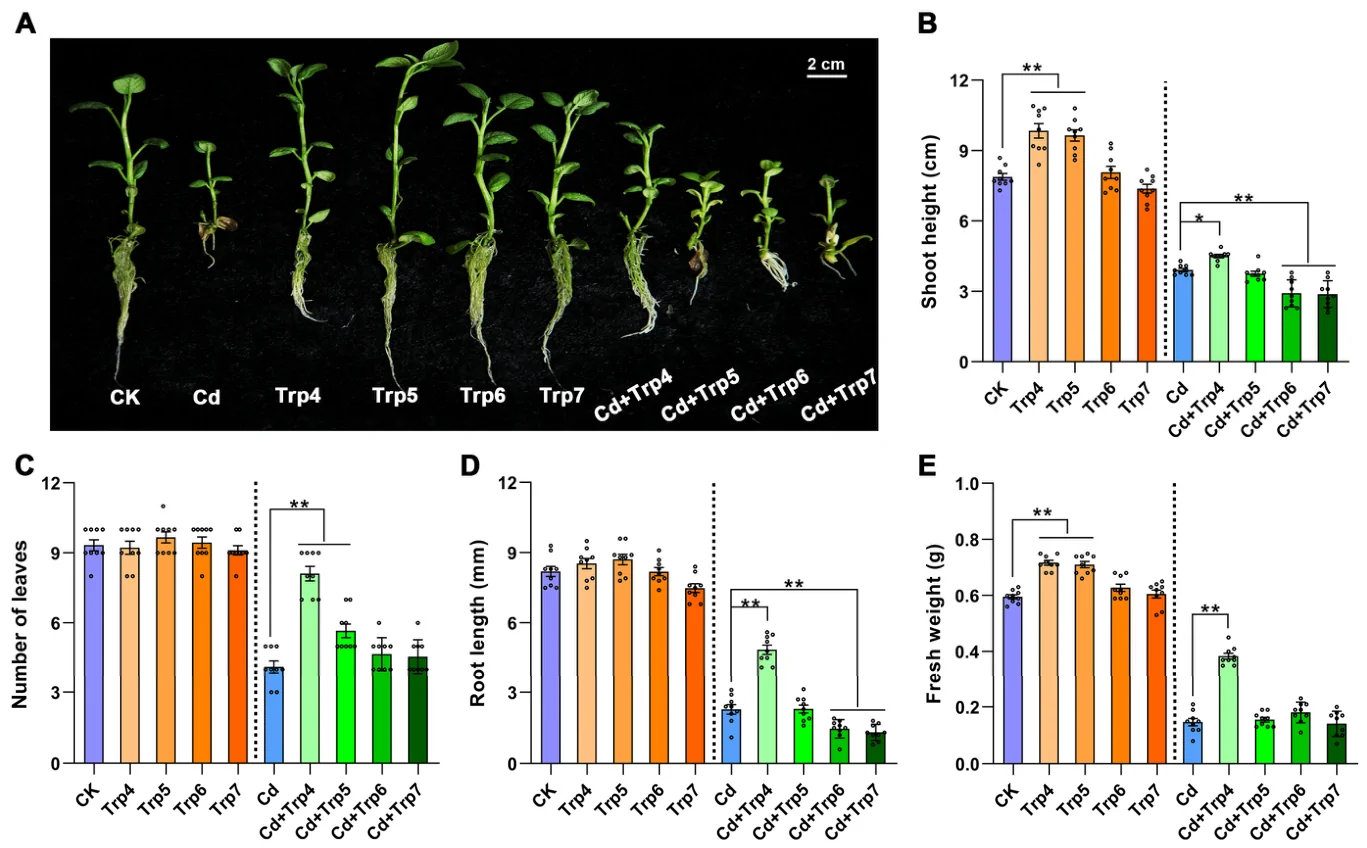
Phenotypic traits of potato plantlets treated with low-concentration Trp under Cd stress. (**A**) Seedling morphology, scale bar = 2 cm. (**B**–**E**) Quantitative measurements of shoot height, leaf number, root length, and fresh weight. Planned pairwise comparisons were performed using independent-samples Student’s *t*-test. Asterisks denote significant differences relative to the corresponding control group (non-Cd treatments vs. CK; Cd-containing treatments vs. Cd-only control): * and ** indicate *p* < 0.05 and *p* < 0.01, respectively. Error bars represent standard error (SE) of biological replicates.

**Figure 4:**
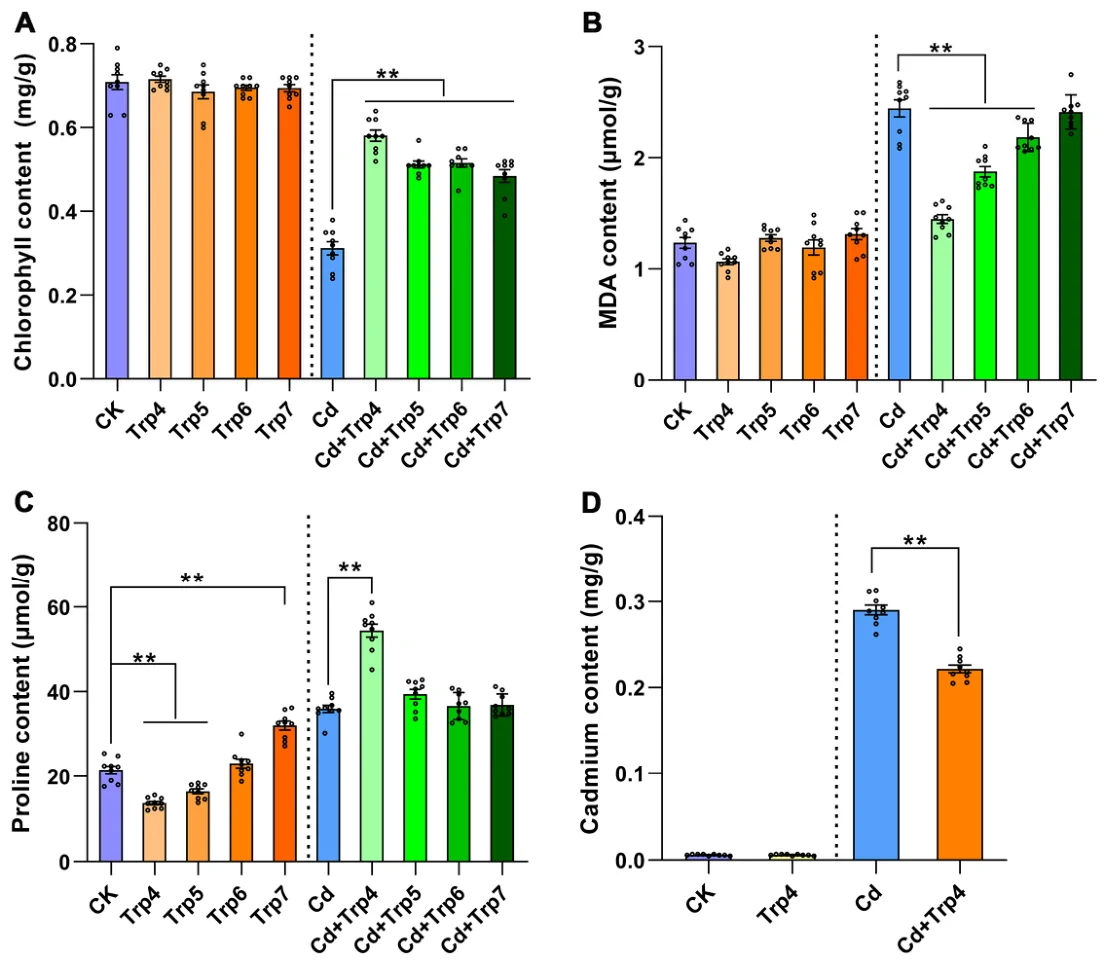
Physiological indexes and Cd accumulation in Cd-stressed potato plantlets treated with low-concentration Trp. (**A**–**C**) Chlorophyll, MDA, and proline contents across Trp4–Trp7 treatments. (**D**) Cd content in CK, Trp4, Cd, and Cd + Trp4 groups. Planned pairwise comparisons were performed using independent-samples Student’s *t*-test. Asterisks denote significant differences relative to the corresponding control group (non-Cd treatments vs. CK; Cd-containing treatments vs. Cd-only control): ** indicate *p* < 0.01. Error bars represent standard error (SE) of biological replicates.

Under Cd-free conditions, Trp treatments caused no toxicity and slightly promoted potato growth. Compared with CK, Trp4 and Trp5 increased shoot fresh weight by 25.32% and 24.18%, respectively, accompanied by moderate improvements in shoot height and leaf number. Root traits were largely maintained or marginally elevated (Figure 3). Chlorophyll and proline levels remained stable across all treatments, confirming the biosafety of low-concentration Trp (Figure 4). These results indicate that although Trp facilitates normal vegetative growth to a certain extent, its growth-promoting effects under normal conditions are relatively moderate, and further evaluation under Cd stress is required to assess its protective capacity.

#### 3.2.2. Mitigation Effects of Trp on Cd-Damaged Morphology and Root Architecture

Cd severely impaired potato growth, reducing shoot height by 54.51%, root length by 77.70%, and fresh weight by 75.37%. Leaf number, root area, and branching also declined markedly by 55.95%, 90.96%, and 93.04%, respectively (Figure 3A–E). Trp supplementation effectively alleviated this inhibition in a dose-dependent manner, with efficacy diminishing as concentration increased from 5 to 20 μM. The 5 μM treatment (Cd + Trp4) showed the strongest recovery, increasing shoot height, total root length, and fresh weight by 27.67%, 180.37%, and 161.36% over Cd alone (Figures S5A and S6A–E). Leaf number, root area, and branching were also substantially restored. Higher Trp concentrations yielded progressively weaker effects on both shoot and root traits.

#### 3.2.3. Trp Modulates Leaf Physiology and Cd Accumulation in Cd-Stressed Plantlets

Cd disrupted photosynthesis and induced oxidative stress in potato leaves, reducing chlorophyll by 55.96% while elevating MDA and proline by 67.49% and 97.95%, respectively (Figure 4A–C). Cd + Trp4 significantly restored chlorophyll to 86.12% above Cd levels, concurrently decreasing MDA by 40.72% and further increasing proline by 51.38%, thereby enhancing antioxidant defense against Cd toxicity. Moreover, 5 μM Trp reduced tissue Cd accumulation by 23.58% (Figure 4D). Higher Trp concentrations exhibited progressively weaker alleviating effects on physiological parameters, consistent with the phenotypic trends observed.

### 3.3. Refined Screening of Low-Concentration MT and Identification of the Optimal Treatment

#### 3.3.1. Normal Growth Performance Under Single MT Treatment

**Figure 5:**
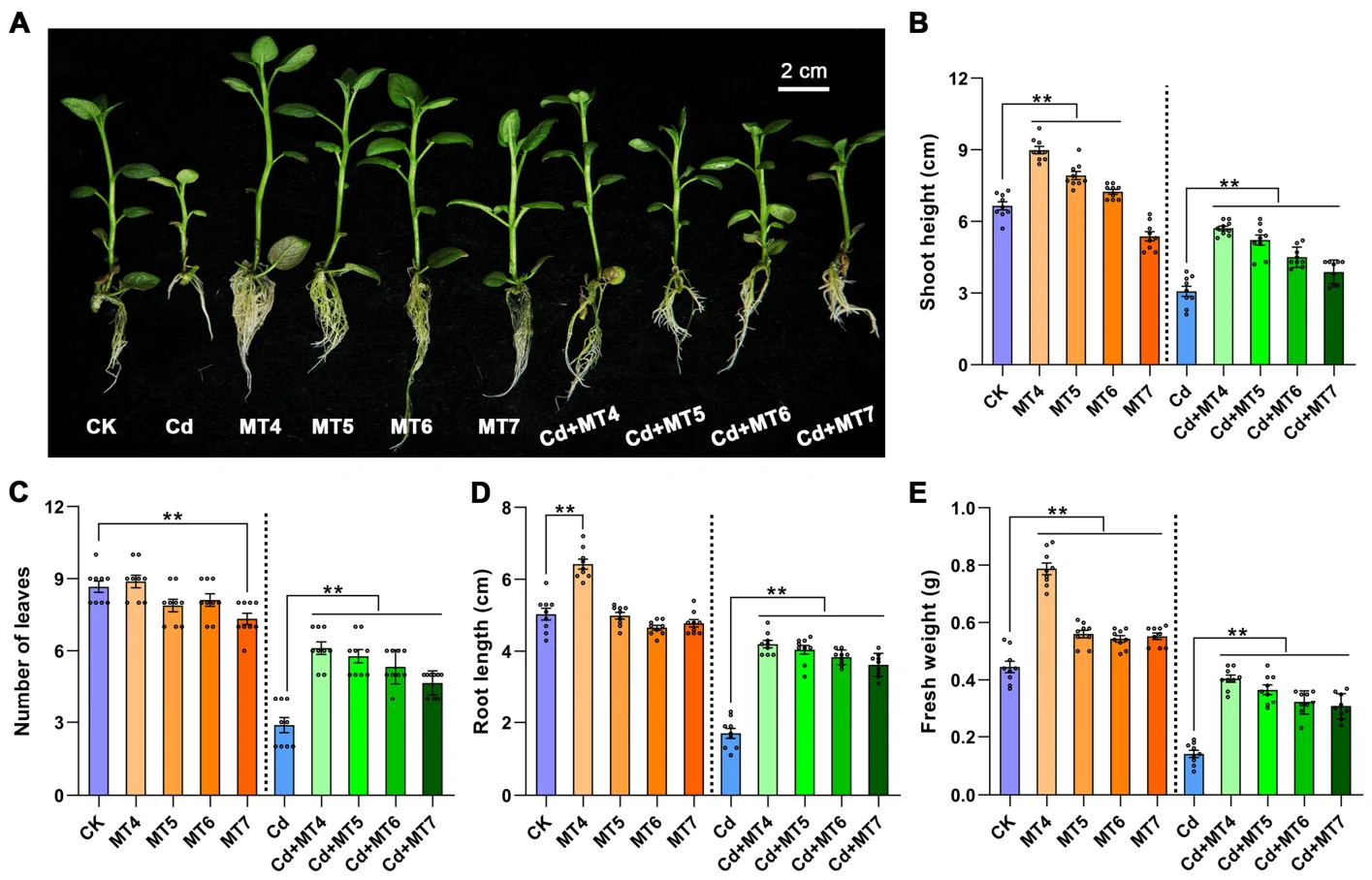
Phenotypic traits of potato plantlets treated with low-concentration MT under Cd stress. (**A**) Seedling morphology, scale bar = 2 cm. (**B**–**E**) Quantitative measurements of shoot height, leaf number, root length, and fresh weight. Planned pairwise comparisons were performed using independent-samples Student’s *t*-test. Asterisks denote significant differences relative to the corresponding control group (non-Cd treatments vs. CK; Cd-containing treatments vs. Cd-only control): ** indicate *p* < 0.01. Error bars represent standard error (SE) of biological replicates.

**Figure 6:**
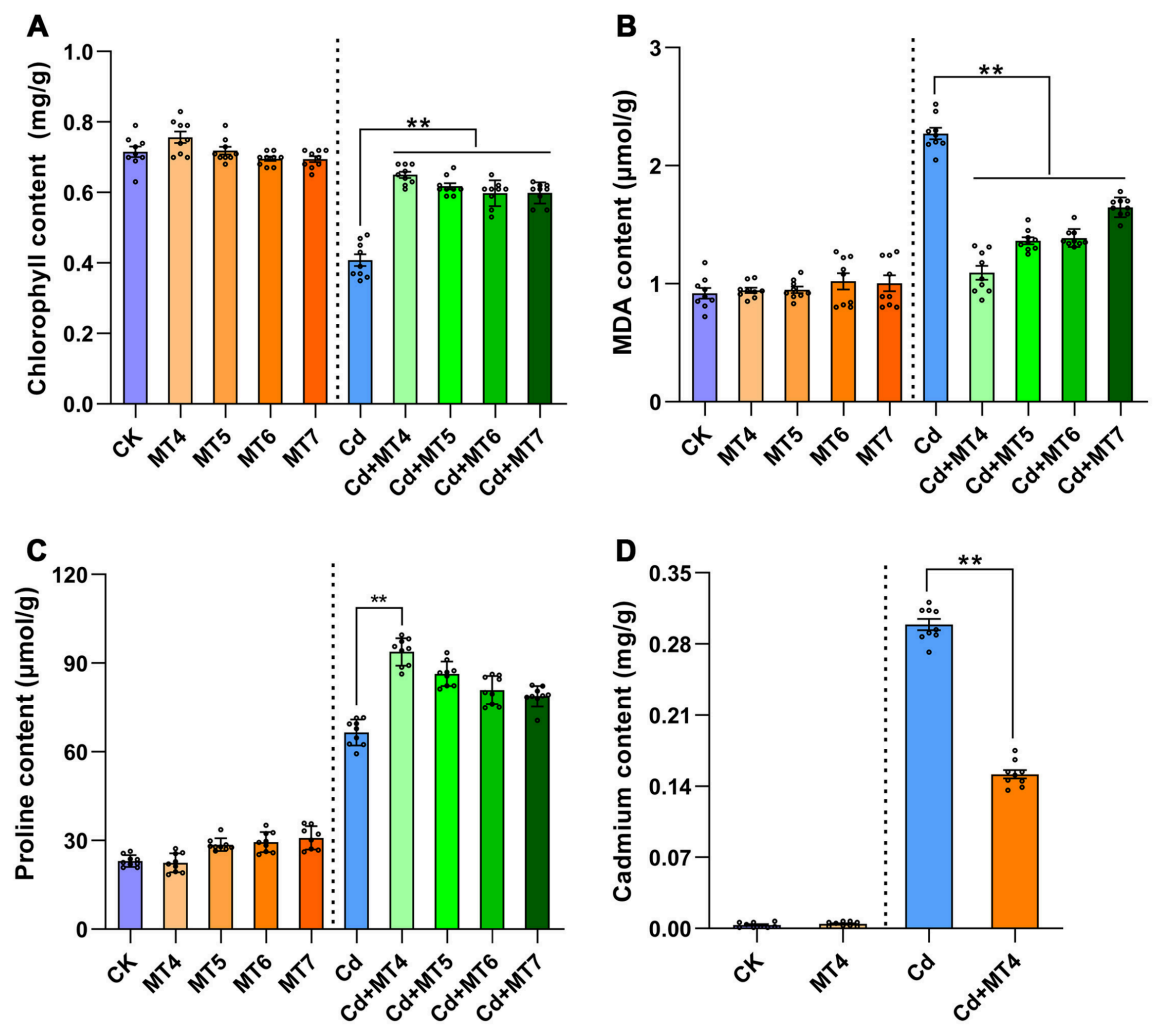
Physiological indexes and Cd accumulation in Cd-stressed potato plantlets treated with low-concentration MT. (**A**–**C**) Chlorophyll, MDA, and proline contents across MT4–MT7 treatments. (**D**) Cd content in CK, MT4, Cd, and Cd + MT4 groups. Planned pairwise comparisons were performed using independent-samples Student’s *t*-test. Asterisks denote significant differences relative to the corresponding control group (non-Cd treatments vs. CK; Cd-containing treatments vs. Cd-only control): ** indicate *p* < 0.01. Error bars represent standard error (SE) of biological replicates.

Under Cd-free conditions, four MT concentrations promoted potato growth without phytotoxicity. The 5 μM treatment (MT4) showed the strongest stimulation, increasing shoot fresh weight by 35.11% compared with CK. Shoot height, leaf number, and root branching were also markedly improved. Higher concentrations (10–20 μM) displayed progressively weaker effects, with a gradual decline in fresh weight and root parameters as MT dosage increased (Figure 5). Chlorophyll, MDA, and proline levels remained stable across all MT-alone treatments, indicating normal physiological status was maintained (Figure 6). These results demonstrate that low-concentration MT is safe for in vitro potato plantlets and effectively facilitates normal vegetative growth, providing a foundation for its protective role under Cd stress.

#### 3.3.2. Mitigation Effects of MT on Cd-Damaged Morphology and Root Architecture

Cadmium stress severely inhibited potato growth and root architecture. Compared with the control, Cd alone reduced shoot height by 53.74%, root length by 66.22%, and shoot fresh weight by 68.33%; root surface area, volume, and branching decreased by 75.36%, 81.92%, and 64.09%, respectively (Figure S5B and Figure 5A–E). Melatonin supplementation effectively alleviated Cd toxicity, with protective efficacy diminishing as concentration increased. The 5 μM treatment (Cd + MT4) exhibited the strongest repair capacity. Relative to Cd alone, Cd + MT4 increased shoot height by 85.56%, total root length by 147.06%, and shoot fresh weight by 186.61%. Leaf number, root surface area, and branching were restored to levels 111.54%, 282.19%, and 149.23% higher than Cd alone (Figure S7A–E). Higher concentrations (10–20 μM) achieved only limited recovery and failed to reverse Cd damage efficiently.

#### 3.3.3. MT Modulates Leaf Physiology and Cd Accumulation in Cd-Stressed Plantlets

Cadmium disrupted photosynthesis and triggered oxidative burst in potato leaves. Cd alone reduced chlorophyll by 43.01% and elevated MDA and proline by 147.25% and 188.61%, respectively (Figure 6A–C). Cd + MT4 recovered chlorophyll to 64% above Cd levels, while decreasing MDA by 51.88% and further increasing proline by 40.99%. The accumulated proline enhanced non-enzymatic antioxidant capacity to scavenge ROS under Cd stress. Moreover, 5 μM of MT reduced Cd deposition by 49.26% compared with Cd alone (Figure 6D). Consistent with phenotype data, the protective effects of MT on physiological parameters and Cd accumulation diminished progressively from 5 μM to 20 μM.

Combining morphological, root structural, and physiological data across the four MT concentrations, 5 μM of melatonin (MT4) was identified as the optimal concentration to alleviate cadmium toxicity in potato plantlets. Combined with the screening outcomes of L-Trp in Section 3.2, MT exhibited more superior mitigation efficiency against cadmium stress at the same low concentration gradient. Therefore, CK, Cd, MT4, and Cd + MT4 (four treatment groups) were selected for subsequent transcriptome sequencing to explore the underlying molecular regulatory mechanism of melatonin-mediated cadmium tolerance.

### 3.4. Evaluation of Transcriptome Sequencing Quality and Sample Repeatability

To investigate the molecular mechanisms underlying MT-mediated alleviation of Cd toxicity in potato, transcriptome sequencing was performed on four groups: CK, Cd, MT, and Cd + MT. A total of 955.80 million raw reads were generated, yielding 946.82 million clean reads after quality filtering. The average Q20 and Q30 values across all samples were 98.78% and 96.07%, respectively, indicating high sequencing quality. The average GC content was 42.47%. Clean reads were mapped to the potato reference genome (DM_1-3_516_R44_v6.1). The overall mapping rate averaged 86.84%, with uniquely mapped reads accounting for 83.87%. Among the mapped reads, 95.68% were located in exonic regions, while 2.58% and 1.74% were located in intronic and intergenic regions, respectively.

**Figure 7:**
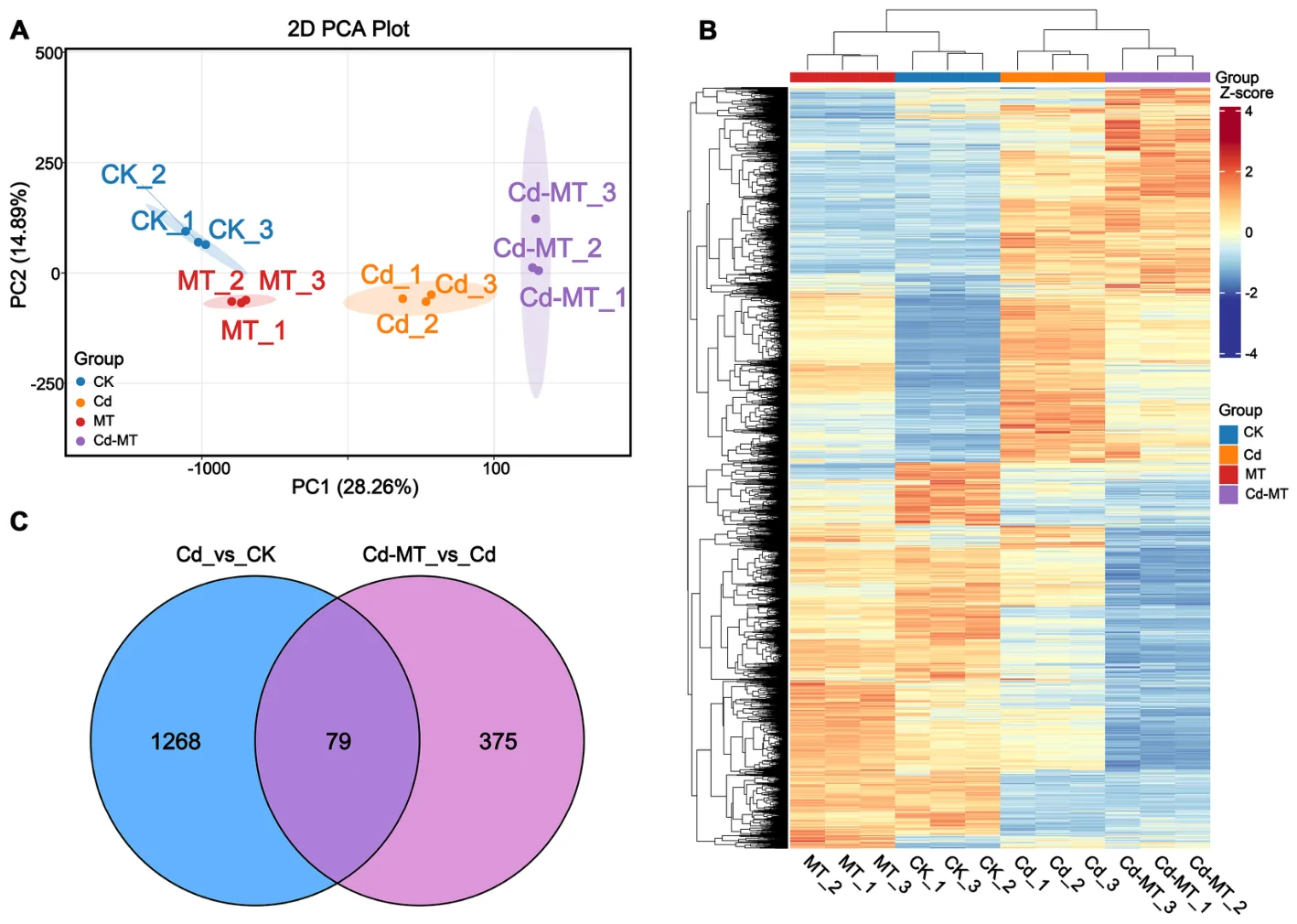
Overall transcriptome variation and differentially expressed gene (DEG) analysis of potato plantlets under melatonin (MT) and cadmium co-treatment. (**A**) Two-dimensional PCA plot showing transcriptional separation of CK, MT, Cd, and Cd-MT groups. (**B**) Hierarchical clustering heatmap of all expressed genes based on Z-score normalized expression values. (**C**) Venn diagram displaying overlapping and specific DEGs identified in Cd vs. CK and Cd-MT vs. Cd comparisons.

Principal component analysis (PCA) was performed to evaluate the global transcriptional relationships among samples. As shown in Figure 7A, the four treatment groups were clearly separated along PC1 (28.26%) and PC2 (14.89%), indicating that both Cd stress and MT treatment induced substantial transcriptomic reprogramming. The CK and MT samples clustered closely, whereas the Cd group exhibited a marked shift along PC1, reflecting strong Cd-induced transcriptional responses. Notably, the Cd + MT group was positioned between CK and Cd, suggesting that MT supplementation partially reversed Cd-induced transcriptomic alterations.

Differentially expressed genes were identified with thresholds of |log_2_FC| > 1 and padj < 0.05. A Venn diagram was constructed to visualize the overlap and specificity of DEGs among the three comparisons (Figure 7B). Venn analysis displayed 1268 DEGs specifically regulated under Cd treatment, 375 DEGs uniquely modulated by melatonin supplementation under Cd stress, and 79 overlapping DEGs responsive to both Cd treatment and melatonin application (Figure 7C). These 79 shared genes were regarded as core candidate genes participating in melatonin-mediated cadmium tolerance in potato.

### 3.5. DEG Screening and Functional Enrichment

**Figure 8:**
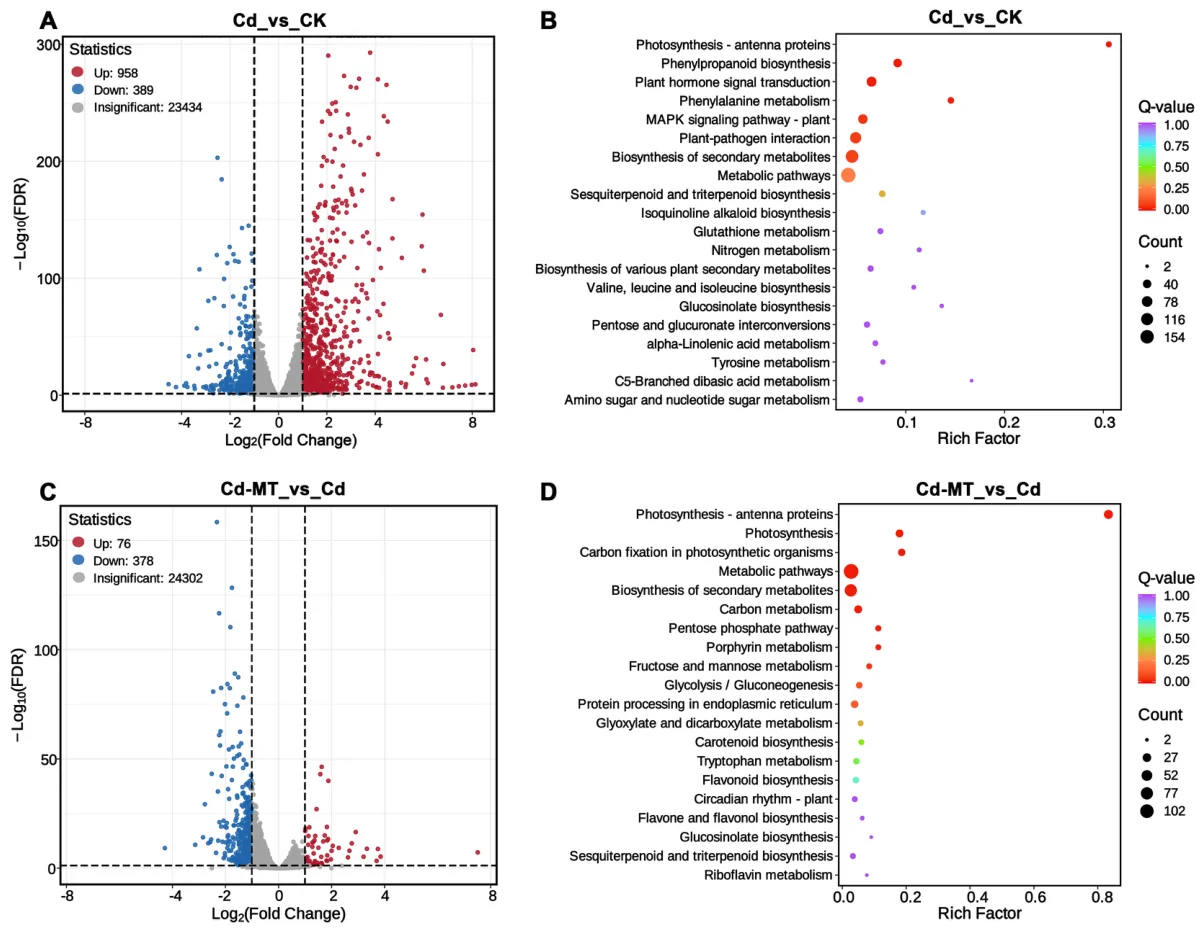
Volcano plots and KEGG enrichment analyses of differentially expressed genes (DEGs) in two comparison groups. (**A**,**B**) Volcano plot and KEGG bubble plot of DEGs for Cd vs. CK. In volcano plot, red dots represent upregulated genes, blue dots represent downregulated genes, and gray dots represent non-significant genes. Bubble size corresponds to the number of enriched genes, and color gradient indicates Q-value. (**C**,**D**) Volcano plot and KEGG enrichment bubble plot of DEGs for Cd-MT vs. Cd.

Differentially expressed genes (DEGs) were screened with thresholds of |log_2_FC| ≥ 1 and FDR < 0.05. In the Cd vs. CK comparison, 1347 DEGs were identified, comprising 958 upregulated and 389 downregulated genes (Figure 8A). In the Cd-MT vs. Cd comparison, 454 DEGs were detected, with 76 upregulated and 378 downregulated genes (Figure 8C). Volcano plots revealed divergent transcriptional reprogramming patterns induced by Cd stress and reversed by MT supplementation.

KEGG enrichment analysis was performed to identify the biological pathways affected by Cd and MT. In Cd vs. CK, the most significantly enriched pathways included photosynthesis-antenna proteins, phenylpropanoid biosynthesis, plant hormone signal transduction, MAPK signaling, and glutathione metabolism. These pathways are closely associated with photosynthetic damage, oxidative stress, and stress signaling activation (Figure 8B). In Cd-MT vs. Cd, MT treatment prominently reversed the enrichment of photosynthesis-related pathways, including photosynthesis, photosynthetic carbon fixation, and porphyrin metabolism (Figure 8D). In addition, multiple carbohydrate metabolic pathways, such as glycolysis, pentose phosphate pathway, and fructose and mannose metabolism, were significantly enriched, suggesting that MT repaired Cd-impaired photosynthetic function and remodeled central carbon metabolism.

GO enrichment analysis further supported these trends. In Cd vs. CK, enriched biological processes included hypoxia response, wound response, ethylene and jasmonic acid signaling, photosystem light harvesting, and oxidoreductase activity. After MT application, GO terms shifted predominantly to photosynthesis, thylakoid membrane organization, photosystem assembly, and carbon fixation (Figure S8A,B).

### 3.6. WGCNA Network Construction and Module–Physiology Correlation

**Figure 9:**
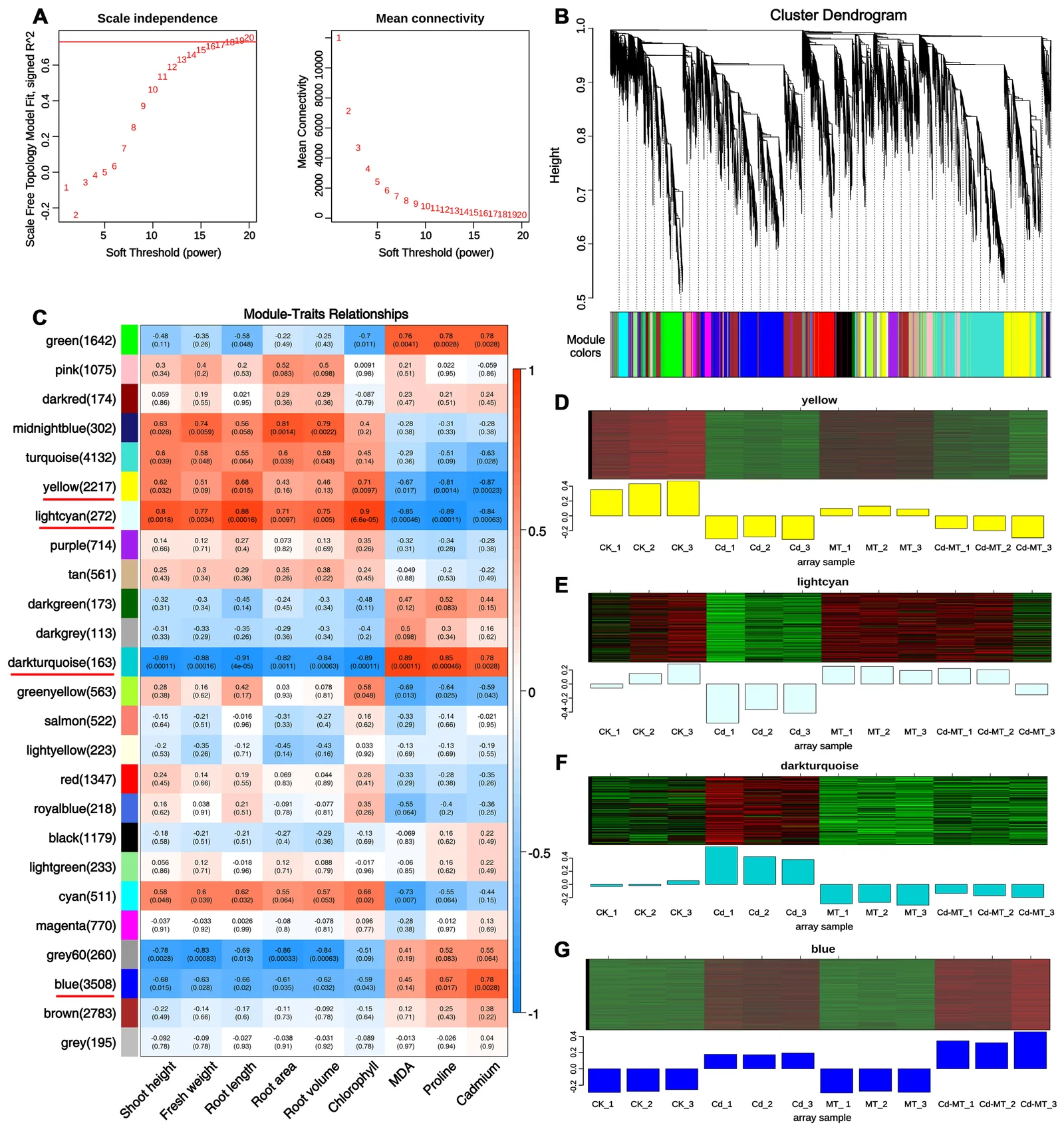
Weighted gene co-expression network analysis (WGCNA) of potato transcriptome responding to melatonin and Cd treatment. (**A**) Screening of optimal soft threshold power based on scale-free topology fitting index and mean connectivity. (**B**) Gene clustering dendrogram and corresponding color-coded co-expression modules. (**C**) Heatmap showing correlation coefficients between 25 gene modules and nine physiological traits. (**D**–**G**) Expression heatmaps of module eigengenes (ME) for four key modules, namely yellow, lightcyan, darkturquoise, and blue, across all samples.

Weighted gene co-expression network analysis (WGCNA) was performed to link transcriptional variation to nine physiological traits, namely shoot height, fresh weight, root length, root area, root volume, chlorophyll content, MDA, proline, and cadmium concentration. An appropriate soft-thresholding power of 18 was selected based on scale independence (R^2^ > 0.85) and mean connectivity criteria to ensure network scale-free topology (Figure 9A). Through hierarchical clustering and dynamic tree cutting, all expressed genes were assigned to 25 distinct co-expression modules (Figure 9B).

Module–trait correlation analysis identified multiple modules significantly associated with physiological phenotypes (Figure 9C). Four representative modules, yellow (2217 genes), lightcyan (272 genes), darkturquoise (163 genes), and blue (3508 genes), exhibited the highest correlation coefficients with Cd accumulation, chlorophyll, MDA, and proline, and were selected for further investigation. The eigengene expression patterns of these four modules across samples are visualized in Figure 9D–G.

The yellow module showed elevated expression in Cd-treated samples and reduced levels in CK and Cd + MT groups, consistent with the accumulation of MDA, proline, and Cd under stress (Figure 9D). The lightcyan module displayed low expression under Cd stress but recovered upon MT treatment, mirroring the restoration of photosynthetic and root growth traits (Figure 9E). The darkturquoise module was highly expressed in CK but suppressed by Cd, with partial recovery after MT application (Figure 9F). The blue module exhibited the lowest expression under Cd stress and was significantly upregulated by MT supplementation, positively correlating with chlorophyll content and root morphological parameters (Figure 9G). These four modules were considered core gene sets governing physiological changes during Cd stress and MT-mediated alleviation, and genes within them were prioritized as candidates for regulating growth, antioxidant homeostasis, and cadmium accumulation.

### 3.7. WGCNA Hub Gene Screening, qRT-PCR Validation, and Regulatory Model Construction

**Figure 10:**
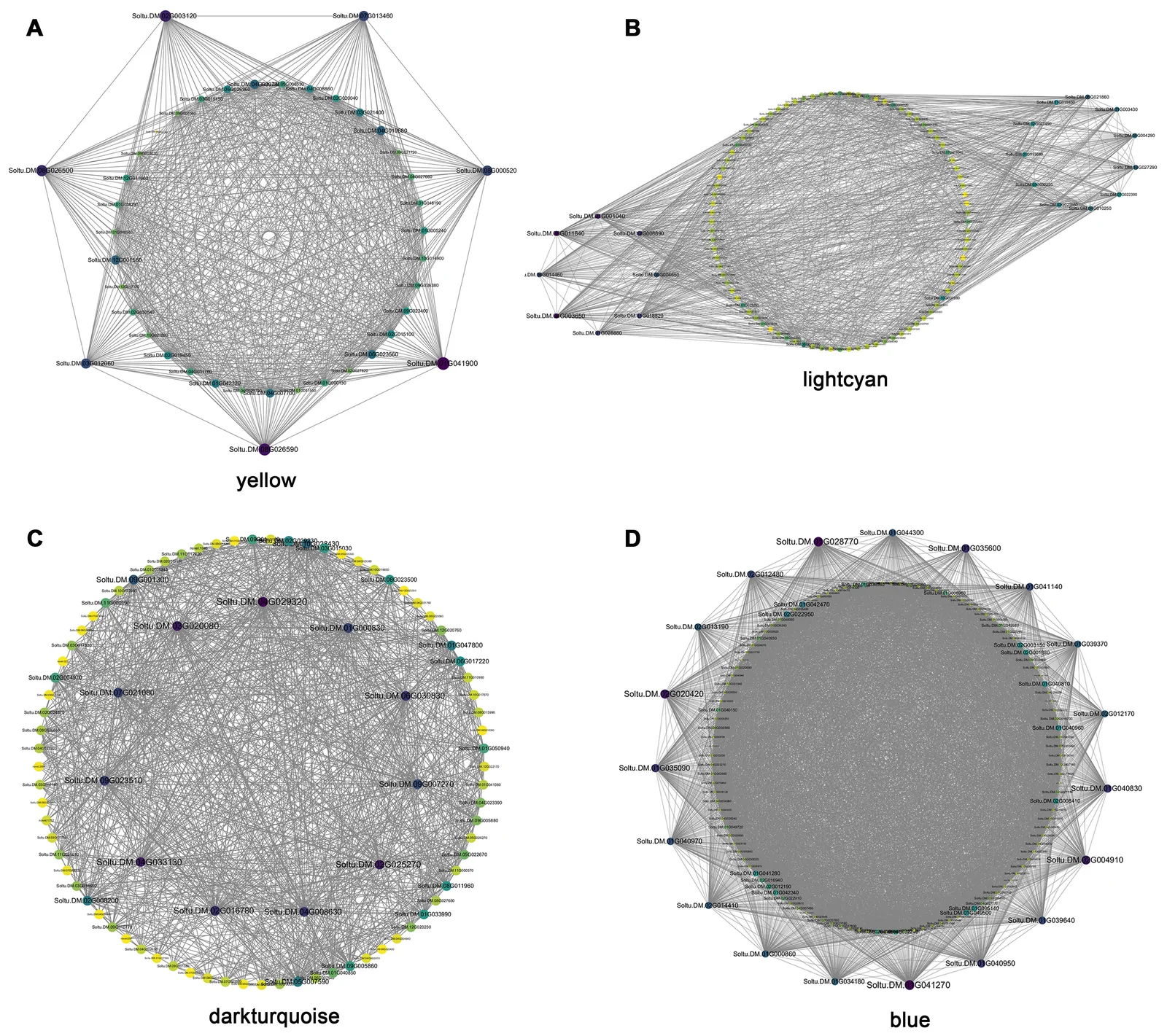
Gene co-expression networks of four key WGCNA modules associated with potato cadmium tolerance: (**A**) yellow module, (**B**) lightcyan module, (**C**) darkturquoise module, and (**D**) blue module. Nodes represent individual genes, and gray connecting lines indicate pairwise gene co-expression relationships. Node colors gradient from yellow (low intramodular connectivity), green-cyan (moderate connectivity), dark-blue (high connectivity) to purple (hub genes with the highest connectivity within the module). Grey edges indicate co-expression relationships between gene pairs.

Weighted gene co-expression network analysis (WGCNA) was conducted to screen physiologically relevant gene modules associated with potato Cd tolerance. Four pivotal co-expression modules, namely yellow, lightcyan, darkturquoise and blue, were identified. Gene co-expression networks of the four modules were visualized (Figure 10), in which nodes represented genes and connecting lines indicated gene expression correlations. Multiple hub genes with high intramodular connectivity were extracted from each module according to gene significance (GS) and module membership (MM) values.

**Figure 11:**
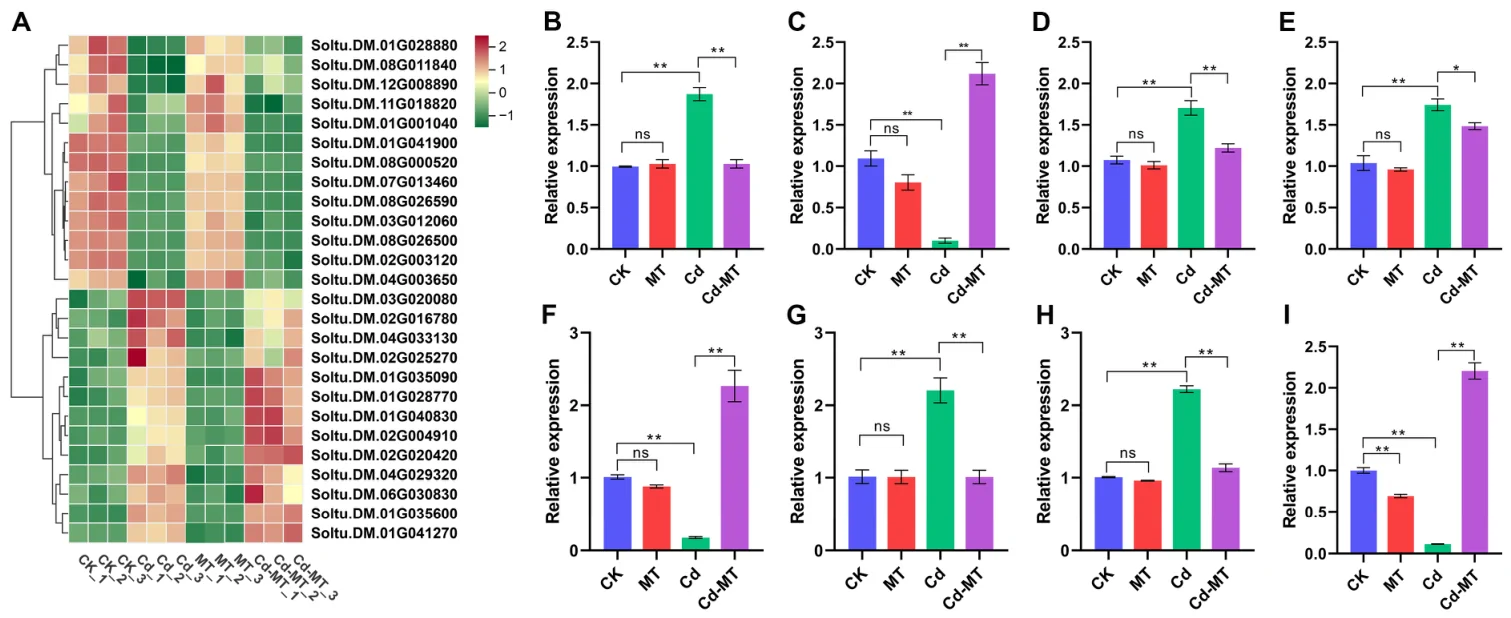
Expression profiling and qRT-PCR validation of candidate hub genes derived from WGCNA modules. (**A**) Heatmap displaying FPKM-based transcript abundances of 26 candidate hub genes across four experimental groups (CK, MT, Cd, and Cd-MT). (**B**–**I**) Quantitative expression results of eight validated hub genes determined via qRT-PCR: *StSAM-2* (Soltu.DM.01G040830), *StCAS1* (Soltu.DM.07G013460), *StSWEET10* (Soltu.DM.03G020080), *StCYP81K2* (Soltu.DM.04G033130), *StSDD1* (Soltu.DM.08G026500), *StHMG1* (Soltu.DM.02G004910), *StANNAT1* (Soltu.DM.04G029320), and *StNRT1.1* (Soltu.DM.08G000520). Planned pairwise comparisons were performed using independent-samples Student’s *t*-test. Asterisks denote significant differences relative to the corresponding control group (non-Cd treatments vs. CK; Cd group vs. CK; Cd-MT group vs. Cd-only control): * and ** indicate *p* < 0.05 and *p* < 0.01, respectively; ns indicates no significant difference. Error bars represent standard error (SE) of biological replicates.

To validate the reliability of the transcriptomic data, eight representative hub genes were selected for qPCR analysis (Figure 11B–I): *StSAM-2* (Soltu.DM.01G040830), *StCAS1* (Soltu.DM.07G013460), *StSWEET10* (Soltu.DM.03G020080), *StCYP81K2* (Soltu.DM.04G033130), *StSDD1* (Soltu.DM.08G026500), *StHMG1* (Soltu.DM.02G004910), *StANNAT1* (Soltu.DM.04G029320), and *StNRT1.1* (Soltu.DM.08G000520). The qPCR results showed consistent expression trends with the RNA-seq FPKM profiles across all four treatments, confirming the robustness of the transcriptome data and the WGCNA-based gene-selection strategy.

**Figure 12:**
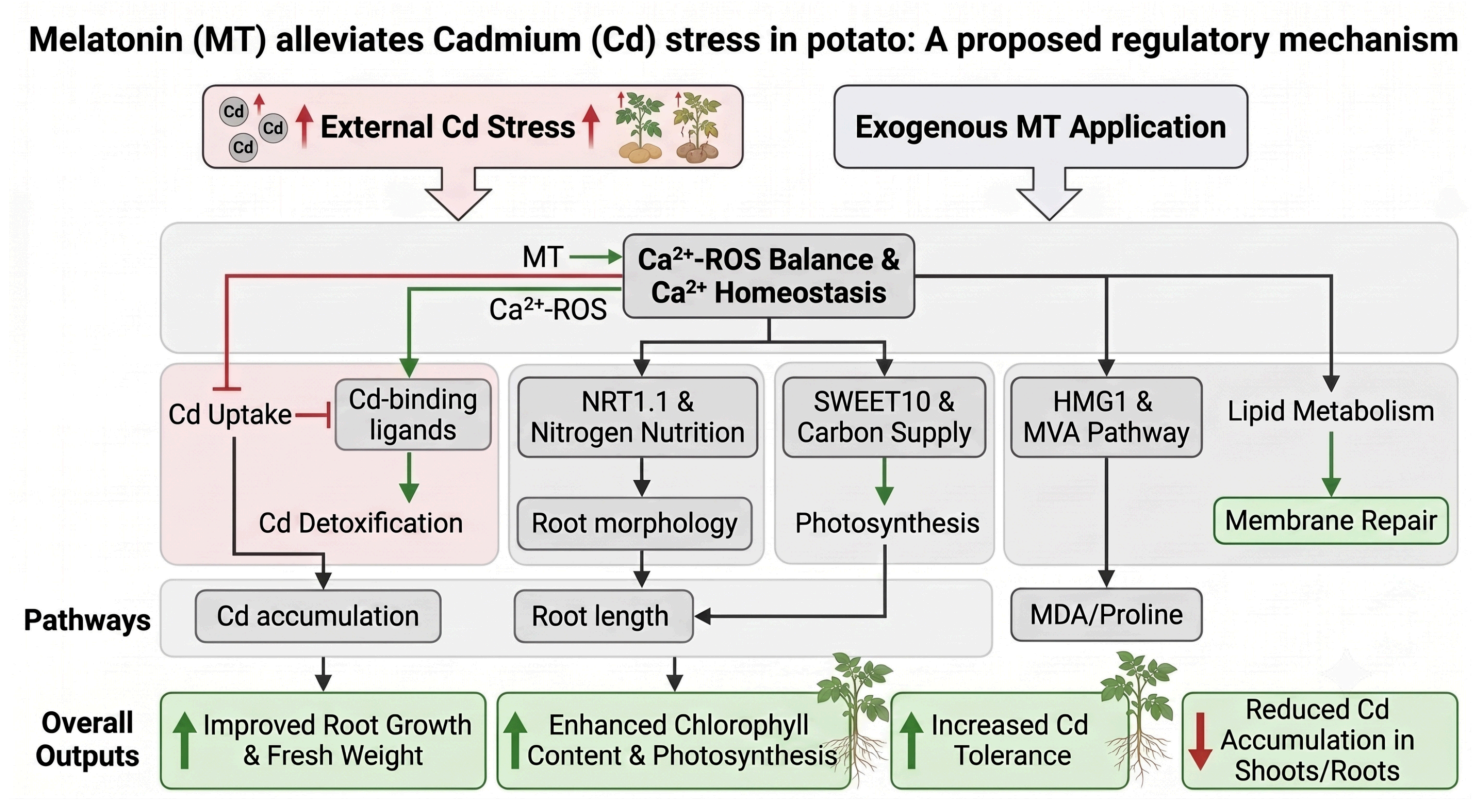
Schematic model illustrating the regulatory network by which exogenous melatonin alleviates cadmium stress in potato.

Functional classification indicated these eight genes participated in five major biological pathways, namely Ca^2+^-ROS homeostasis (*StANNAT1*), nitrogen transport and root development (*StNRT1.1*), carbon allocation and photosynthesis (*StSWEET10*), MVA pathway and membrane repair (*StHMG1* and *StCYP81K2*), as well as hormone metabolism and photosystem stability (*StCAS1*, *StSAM-2*, and *StSDD1*). Combined with physiological data and transcriptomic findings, a comprehensive regulatory model was proposed to illustrate how exogenous melatonin alleviates Cd toxicity in potato (Figure 12). MT balanced cellular Ca^2+^-ROS status, restricted root Cd uptake, optimized carbon and nitrogen nutrition distribution, restored photosynthetic capacity and root morphology, and ultimately reduced Cd accumulation and oxidative damage in potato plants.

## 4. Discussion

Cadmium contamination in agricultural soils poses persistent risks to crop production and food safety. This study systematically compared three exogenous regulators, namely NAC, Trp, and melatonin, on their effects of mitigating cadmium toxicity in potato. Two rounds of concentration gradient screening were carried out, combined with physiological detection, transcriptome sequencing and WGCNA analysis. The results verified that 5 μM of melatonin exerted the most remarkable protective effects. Melatonin restored root morphology, improved photosynthetic capacity, reduced cadmium accumulation, and recovered cellular redox balance. Transcriptomic and co-expression network analyses identified four core gene modules and eight hub genes. These genes regulate calcium–ROS homeostasis, carbon and nitrogen distribution, and membrane repair, constructing a multi-pathway model to interpret melatonin-mediated cadmium tolerance in potato.

### 4.1. Differential Alleviation Efficiency of NAC, Trp, and MT

Among the three tested regulators, NAC showed no observable mitigation of Cd toxicity within the concentration range and experimental conditions examined in this study. This result was unexpected, given that NAC is known to serve as a precursor of glutathione (GSH) in other plant systems, where GSH acts as a central antioxidant and heavy-metal chelator [23]. However, GSH levels were not directly measured in the present study, and this mechanistic link remains speculative. Several possible explanations may account for this inefficacy. First, the tested NAC concentrations (125–500 μM) might have been suboptimal for potato, although they fell within ranges effective in other species [24]. Notably, this medium-to-high concentration range may have missed its potential beneficial low-dose window, since NAC can display a hormetic biphasic response and trigger pro-oxidant stress at elevated concentrations. Second, NAC uptake or intracellular conversion to GSH may be limited in potato roots, constraining its bioavailability. Third, although not directly tested in this study, NAC has been reported to exert pro-oxidant effects under certain conditions, potentially aggravating Cd-induced ROS production rather than suppressing it [25]. Alternatively, it is possible that the potato Cd stress response may rely more on GSH-independent pathways, such as phytochelatin synthesis or metallothionein regulation, which NAC supplementation cannot effectively activate. These possibilities warrant further investigation using targeted metabolomics to track NAC and GSH dynamics in planta, and future screening should include ultra-low NAC concentrations (5–50 μM) to fully evaluate its protective potential.

In contrast, both Trp and MT exhibited clear concentration-dependent protective effects, with the lowest tested concentrations (5–25 μM) showing the strongest activity. This inverse dose–response pattern is noteworthy. It suggests that higher concentrations of these endogenous signaling molecules may trigger feedback inhibition or off-target effects that compromise their protective functions. For Trp, elevated concentrations could shift metabolic flux toward auxin biosynthesis via the indole-3-acetamide pathway, potentially inducing excessive ethylene production and growth retardation under stress conditions [26,27]. For MT, high-dose pro-oxidant effects have been documented in several plant systems, where excessive MT may act as a pro-oxidant by participating in Fenton-type reactions or by overstimulating ROS-producing enzymes such as NADPH oxidases [28]. Additionally, high concentrations of exogenous MT may saturate membrane receptors or disrupt endogenous MT signaling homeostasis, attenuating its regulatory efficacy [29]. This biphasic effect refers to stimulation at low doses and inhibition at high doses, which aligns with the hormetic response widely detected in phytohormones and signal regulators [30].

The stronger performance of MT over Trp, particularly at the same low concentration (5 μM), may be attributed to their biosynthetic relationship. Trp serves as the precursor for MT biosynthesis via the tryptophan decarboxylase (TDC), tryptamine 5-hydroxylase (T5H), and serotonin N-acetyltransferase (SNAT) pathway [31]. Our observation that Trp exhibited moderate protective effects while MT showed superior efficacy suggests that the conversion efficiency from Trp to MT under Cd stress may be limited in potato. Exogenous MT bypasses this rate-limiting conversion step, directly providing the active molecule to target tissues. This interpretation is supported by the upregulation of *StSAM-2* (S-adenosylmethionine synthase) in our WGCNA analysis, as SAM is a critical methyl donor in MT biosynthesis, implicating enhanced MT biosynthetic capacity in Cd-tolerant plants.

### 4.2. MT-Mediated Restoration of Photosynthesis and Carbon–Nitrogen Metabolism

Our transcriptomic analysis revealed that MT treatment significantly reversed Cd-induced suppression of photosynthesis-related pathways, including photosynthesis-antenna proteins, photosynthetic carbon fixation, and porphyrin metabolism. This is consistent with the physiological data showing that Cd + MT4 restored chlorophyll content to 64% above Cd alone and improved root and shoot biomass.

The upregulation of *StSWEET10* in the Cd + MT group is particularly noteworthy. SWEET family transporters mediate sugar efflux from source to sink tissues, playing essential roles in carbon partitioning under stress conditions [32]. Cadmium stress typically reduces photosynthetic carbon fixation and restricts sugar transport to roots, impairing root development and nutrient uptake [33]. MT-mediated induction of *StSWEET10* may enhance sucrose translocation from leaves to roots, providing both energy and carbon skeletons for root growth and Cd detoxification (e.g., phytochelatin synthesis). This coordinated regulation of carbon allocation represents a previously unrecognized mechanism of MT-conferred Cd tolerance.

Simultaneously, MT treatment enriched carbohydrate metabolism pathways, including glycolysis, the pentose phosphate pathway, and fructose and mannose metabolism. These pathways supply reducing power (NADPH) and metabolic intermediates for GSH regeneration and ROS scavenging [34]. The pentose phosphate pathway, in particular, produces NADPH required for the ascorbate–glutathione cycle, a major antioxidant system that detoxifies H_2_O_2_ in chloroplasts [35]. MT-mediated remodeling of central carbon metabolism thus couples photosynthetic recovery with enhanced antioxidant capacity, forming a self-reinforcing loop that sustains redox balance under Cd stress.

In addition, the upregulation of *StNRT1.1* (a dual-affinity nitrate transporter) in Cd + MT plants suggests improved nitrogen uptake and assimilation. Nitrate reduction consumes NADPH and produces nitric oxide (NO), a signaling molecule that can enhance Cd tolerance by modulating antioxidant enzyme activities and metal transporter expression [36]. The coordinated upregulation of carbon and nitrogen metabolic genes indicates that MT treatment may re-establishes metabolic homeostasis at the whole-plant level, rather than simply activating a single stress-defense pathway.

### 4.3. Ca^2+^-ROS Signaling and Antioxidant Defense as Core MT Targets

A central finding of this study is the identification of MT-modulated pathways related to Ca^2+^-ROS homeostasis. Cadmium stress is known to disrupt cytosolic Ca^2+^ signaling by displacing Ca^2+^ from binding sites and activating Ca^2+^-permeable channels, leading to sustained [Ca^2+^]cyt elevation and downstream ROS burst [37]. The hub gene *StANNAT1* (annexin), which we validated by qPCR, encodes a Ca^2+^-dependent phospholipid-binding protein that functions as a Ca^2+^-permeable transporter and ROS sensor in plants [38]. Annexins are emerging as key players in abiotic stress tolerance, linking Ca^2+^ signals to ROS production and antioxidant gene expression [39,40]. MT-induced upregulation of *StANNAT1* may enhance Ca^2+^ buffering capacity and dampen Cd-triggered Ca^2+^ spikes, thereby preventing excessive ROS generation.

The MVA pathway gene *StHMG1* (3-hydroxy-3-methylglutaryl-CoA reductase) and the cytochrome P450 gene *StCYP81K2* were also identified as MT-responsive hubs. The MVA pathway produces isoprenoid precursors for sterols, brassinosteroids, and ubiquinone, all of which contribute to membrane integrity and electron transport chain function [41]. Cytochrome P450 enzymes are involved in the oxidative metabolism of xenobiotics and endogenous signaling molecules [42,43]. *StCYP81K2* upregulation by MT may indicate enhanced detoxification of lipid peroxidation products, protecting membrane structures from oxidative damage induced by Cd.

The downregulation of MDA and upregulation of proline in Cd + MT plants are consistent with enhanced antioxidant defense. Proline acts as a compatible osmolyte, an ROS scavenger, and a molecular chaperone that stabilizes proteins and membranes under stress [44]. MT treatment further elevated proline beyond Cd-induced levels, suggesting that MT potentiates the endogenous proline accumulation pathway (likely via Δ^1^-pyrroline-5-carboxylate synthetase, P5CS), rather than merely passively responding to stress.

Our WGCNA results showed that the blue module, which was positively correlated with chlorophyll and root traits, contained genes significantly upregulated by MT. The eigengene pattern of the blue module presents the lowest expression under Cd and marked recovery upon MT, strongly suggesting that this module represents the core transcriptional program governing MT-mediated photosynthetic recovery. Functional enrichment of blue module genes in photosynthesis and carbon fixation pathways further supports this interpretation.

### 4.4. MT Reduces Cd Uptake and Accumulation

Cd accumulation in Cd + MT4 plants was reduced by 49.26% compared with Cd alone. This reduction is unlikely to result solely from dilution due to increased biomass, as Cd concentration (on a tissue weight basis) was significantly lower. Instead, MT may directly or indirectly regulate Cd transport systems. Candidate transporters include NRAMP (natural resistance-associated macrophage protein), ZIP (zinc-regulated transporter/iron-regulated transporter-like protein), and HMA (heavy-metal ATPase) families, which are responsible for Cd uptake, xylem loading, and vacuolar sequestration [45]. Although our transcriptomic analysis did not identify a single dominant transporter gene, the coordinated downregulation of multiple Cd uptake-associated genes within the yellow module suggests that MT suppresses the Cd influx system.

Alternatively, MT may promote Cd sequestration in root vacuoles via upregulation of vacuolar transporters such as MTP (metal tolerance protein) or ABC (ATP-binding cassette) transporters, reducing Cd translocation to shoots. The observation that Cd + MT plants showed greater root biomass and root surface area while also showing lower Cd concentration indicates that MT may enhance the root’s capacity to retain Cd without inducing toxicity, effectively creating a “Cd trap” that limits shoot accumulation [46,47].

It should be noted, however, that, in the present study, Cd content was measured in whole plantlets rather than separately in roots and shoots. This is because our experimental design focused on seedling-stage screening to rapidly identify the optimal MT concentration, rather than long-term whole-growth-period cultivation. As a result, we are unable to distinguish whether the observed 49.26% reduction in Cd accumulation was primarily due to decreased root uptake or enhanced root-to-shoot translocation restriction. For potato, a crop where the edible harvest (tubers) develops underground, this distinction is of particular agronomic relevance. Future studies extending the treatment period to the full growth cycle, with separate Cd measurements in roots, shoots, and tubers, are warranted to determine the precise spatial allocation mechanisms underlying MT-mediated Cd restriction.

### 4.5. Limitations and Future Perspectives

Several limitations of this study should be acknowledged. First, the qPCR validation was performed on a limited set of eight hub genes; broader validation using additional independent biological replicates or independent experiments would strengthen the conclusions. Second, although we identified candidate hub genes and pathways, functional characterization via gene knockout or overexpression is needed to establish causality. Third, and most importantly, the present study was conducted under controlled in vitro conditions using sterile potato plantlets on solid MS medium. This system, while necessary for obtaining clean and reproducible transcriptomic data, does not fully replicate the complexity of field soils. In actual agricultural settings, soil physicochemical properties, microbial communities, organic matter binding, and environmental fluctuations may all influence the stability, bioavailability, and root uptake of exogenous melatonin. Therefore, our findings should be interpreted as a molecular proof of concept for melatonin-mediated Cd tolerance in potato, rather than an immediate, directly transferable field recommendation. Field trials with soil-applied MT are essential to validate the practical applicability of these findings before large-scale agricultural adoption. In addition, the actual concentrations of MT, Trp, and NAC in the culture medium were not analytically verified after filter sterilization and addition to the cooled medium. Although the observed concentration-dependent responses support their stability and bioavailability, future studies could benefit from HPLC-based verification to confirm the nominal concentrations.

From a mechanistic perspective, the interplay between MT, Ca^2+^ signaling, ROS dynamics, and metabolic reprogramming warrants deeper investigation using spatiotemporal imaging, single-cell transcriptomics, and pharmacological approaches. The specific roles of *StANNAT1*, *StSWEET10*, and *StNRT1.1* in MT-conferred Cd tolerance are promising avenues for future functional studies.

## 5. Conclusions

In summary, this study demonstrates that exogenous MT, applied at 5 μM, optimally alleviates Cd toxicity in potato by orchestrating a multi-target regulatory network. MT may restore redox balance, enhance photosynthetic carbon assimilation, remodel carbon–nitrogen metabolism, reduce Cd uptake, and promote root development. The identification of eight hub genes involved in Ca^2+^-ROS signaling, membrane repair, and nutrient transport provides a molecular framework for understanding MT actions. These findings advance our mechanistic knowledge of MT-mediated heavy-metal tolerance and deliver a laboratory-based proof of concept for melatonin-enabled Cd resistance. Given the antioxidant-centric mechanisms uncovered, MT represents a promising candidate to mitigate Cd-induced oxidative damage, and further research will be required to translate these laboratory observations toward sustainable potato production in Cd-contaminated agricultural soils.

## Data Availability

The raw transcriptomic data supporting this study were deposited in the NCBI Sequence Read Archive (SRA) under BioProject accession number PRJNA1527097.

## Supplementary Materials

The following supporting information can be downloaded at https://www.mdpi.com/article/10.3390/antiox15091180/s1, Figure S1. Root morphological phenotypes of potato plantlets under cadmium stress following treatments with three exogenous regulators; Figure S2. Root architecture traits of potato plantlets exposed to Cd stress and treated with NAC at three concentrations; Figure S3. Root architecture traits of potato plantlets exposed to Cd stress and treated with MT at three concentrations; Figure S4. Root architecture traits of potato plantlets exposed to Cd stress and treated with Trp at three concentrations; Figure S5. Root morphological phenotypes of potato plantlets after treatment with low-concentration tryptophan (Trp) and melatonin (MT) under cadmium stress. Figure S6. Root morphological traits of potato plantlets exposed to Cd stress supplemented with four low concentrations of melatonin (MT); Figure S7. Root morphological traits of potato plantlets exposed to Cd stress supplemented with four low concentrations of tryptophan (Trp); Figure S8. GO-enrichment bubble analysis of differentially expressed genes (DEGs) in two comparison groups. Table S1. Sequences of primers in RT-qPCR.
