# Supplementary material for: Melatonin Attenuates Cadmium-Induced Oxidative Stress in Potato by Remodeling Redox Homeostasis and Activating Multi-Pathway Antioxidant Networks
Source: Antioxidants (Basel). 2026 Sep 17;15(9):1180. doi: 10.3390/antiox15091180 (PMC13603471; doi:10.3390/antiox15091180)
Supplement: Supplementary file 1 [file antioxidants-15-01180-s001.zip › antioxidants-4507734-supplementary.pdf]

Supporting Information

Melatonin Attenuates Cadmium-Induced Oxidative Stress in Potato by Remodeling Redox Homeostasis and Activating Multi-Pathway Antioxidant Networks

Panfeng Yao [1,2], Junmei Cui [1], Wenlin Li [3], Jianguo Wei [1], Ying Wang [1], Chao Sun [1,2], Zhenzhen Bi [1,2], Zhen Liu [1,2], Han Wang [2], Yuhui Liu [1,2,*], Jiangping Bai [4,*]

[1] State Key Laboratory of Aridland Crop Science, Gansu Agricultural University, Lanzhou 730070, China; yaopf@gsau.edu.cn (P.Y.); cuijm@gsau.edu.cn (J.C.); 1073325121094@st.gsau.edu.cn (J.W.); 1073325121103@st.gsau.edu.cn (Y.W.); sunc@gsau.edu.cn (C.S.); bizz@gsau.edu.cn (Z.B.); liuzhen@gsau.edu.cn (Z.L.)

[2] College of Agronomy/Seed Industry Research Institute of Gansu Provincial University, Gansu Agricultural University, Lanzhou 730070, China; wh@gsau.edu.cn

[3] College of Horticulture, Gansu Agricultural University, Lanzhou 730070, China; liwenl@gsau.edu.cn

[4] School of Materials Science and Engineering, Lanzhou Institute of Technology, Lanzhou 730070, China

* Correspondence: lyhui@gsau.edu.cn (Y.L.); jiangpingbai@yahoo.com (J.B)

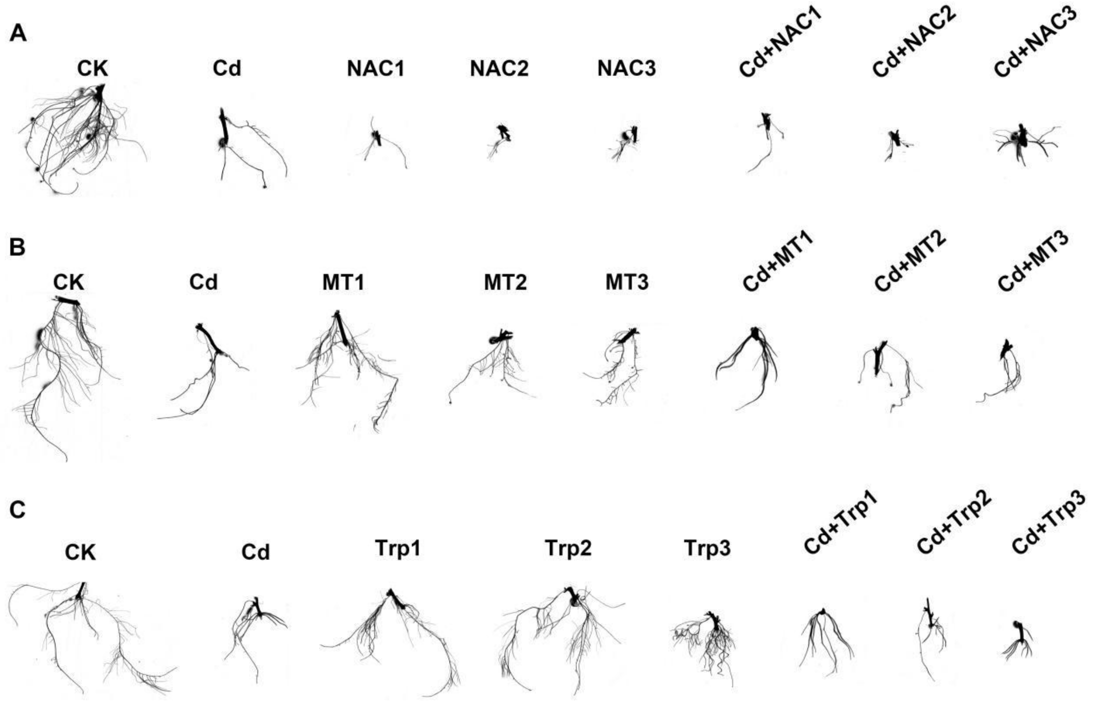

Figure S1. Root morphological phenotypes of potato plantlets under cadmium stress following treatments with three exogenous regulators. (A-C) Root-scan images of plantlets treated with N-acetyl-L-cysteine (NAC), melatonin (MT), and tryptophan (Trp), respectively. CK, control; Cd, cadmium-only treatment; NAC1-NAC3, three concentrations of NAC; MT1-MT3, three concentrations of melatonin; Trp1-Trp3, three concentrations of tryptophan; Cd+NAC1-Cd+NAC3, Cd stress combined with three NAC concentrations; Cd+MT1-Cd+MT3, Cd stress combined with three melatonin concentrations; Cd+Trp1-Cd+Trp3, Cd stress combined with three tryptophan concentrations.

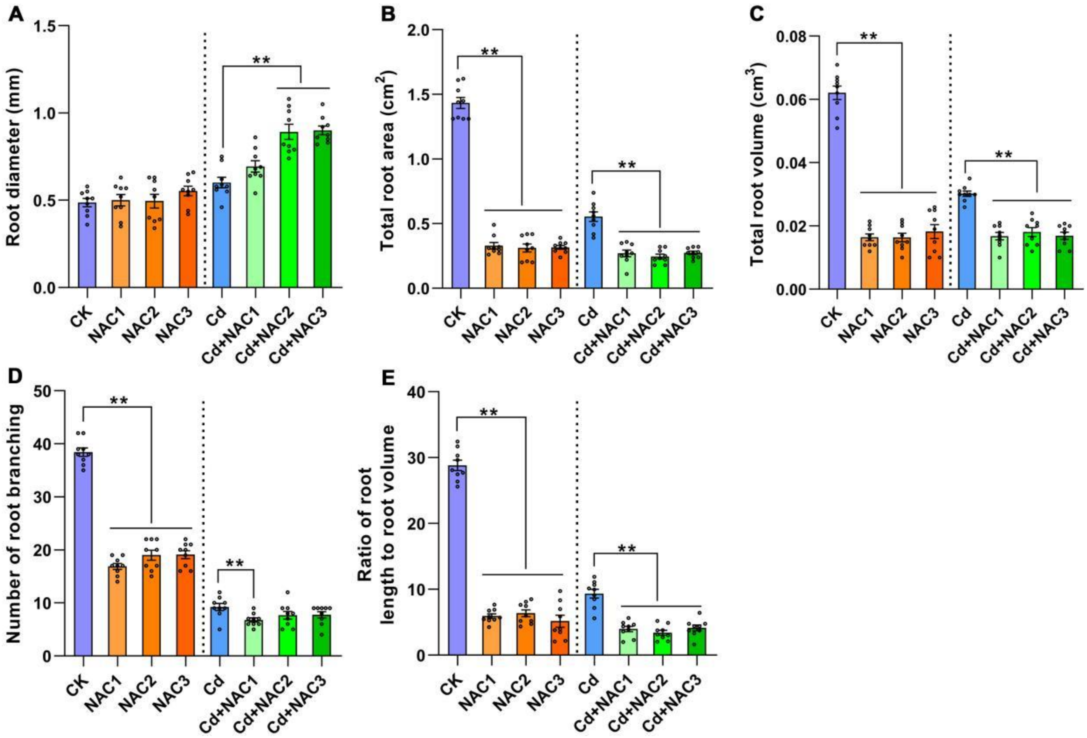

Figure S2. Root architecture traits of potato plantlets exposed to Cd stress and treated with NAC at three concentrations. (A–E) Root diameter, total root area, total root volume, number of root branching, and ratio of root length to root volume. Comparisons between selected pairs of treatments were conducted using Student's t-test. ** indicate significant differences at $P < 0.01$. Error bars represent standard error (SE) of biological replicates.

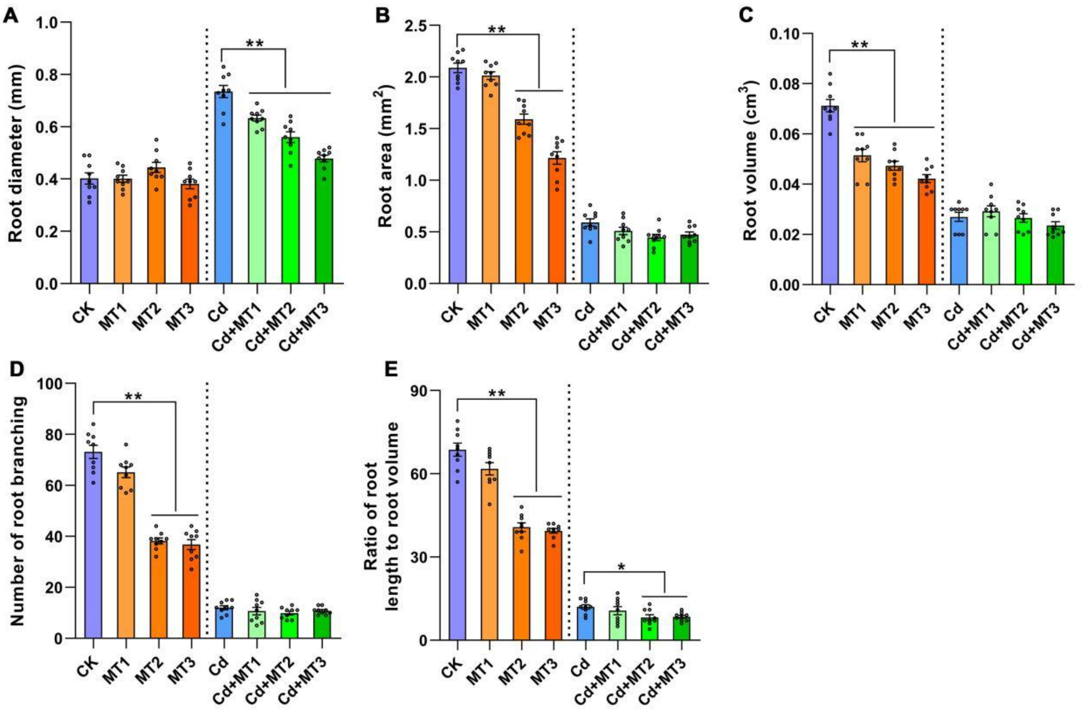

Figure S3. Root architecture traits of potato plantlets exposed to Cd stress and treated with MT at three concentrations. (A–E) Root diameter, total root area, total root volume, number of root branching, and ratio of root length to root volume. Comparisons between selected pairs of treatments were conducted using Student's t-test. ** indicate significant differences at P < 0.01. Error bars represent standard error (SE) of biological replicates.

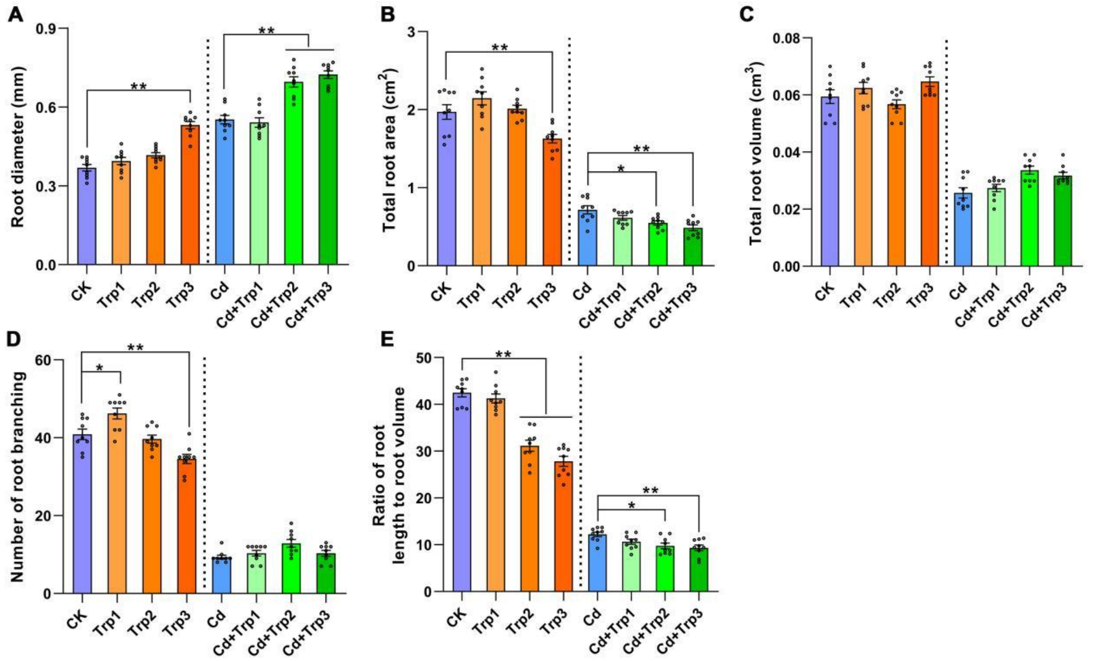

Figure S4. Root architecture traits of potato plantlets exposed to Cd stress and treated with Trp at three concentrations. (A–E) Root diameter, total root area, total root volume, number of root branching, and ratio of root length to root volume. Comparisons between selected pairs of treatments were conducted using Student's t-test. ** indicate significant differences at $P < 0.01$. Error bars represent standard error (SE) of biological replicates.

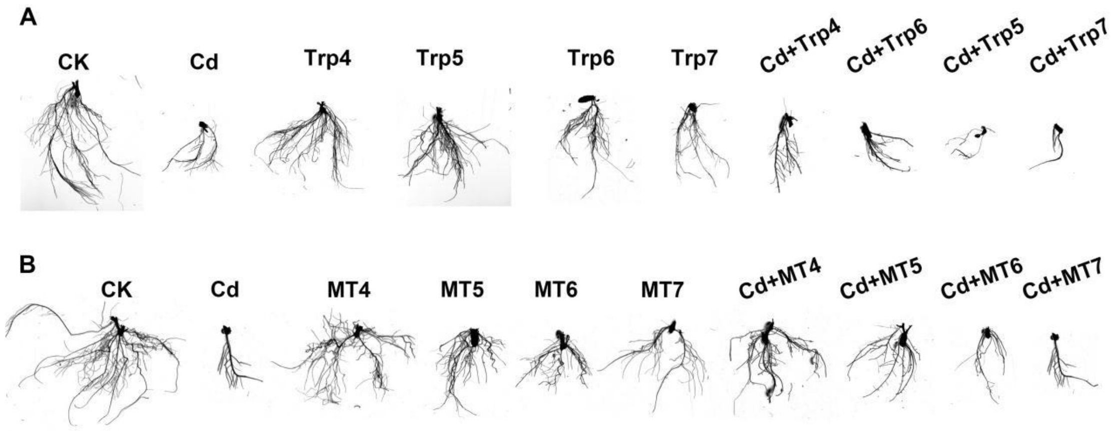

Figure S5. Root morphological phenotypes of potato plantlets after treatment with low-concentration tryptophan (Trp) and melatonin (MT) under cadmium stress. (A) Root-scan images of potato plantlets treated with four concentrations of tryptophan; (B) Root-scan images of potato plantlets treated with four concentrations of melatonin. CK, control group; Cd, cadmium-only treatment; Trp4-Trp7, four low concentrations of tryptophan; MT4-MT7, four low concentrations of melatonin; Cd+Trp4-Cd+Trp7, Cd stress combined with corresponding Trp concentrations; Cd+MT4-Cd+MT7, Cd stress combined with corresponding MT concentrations.

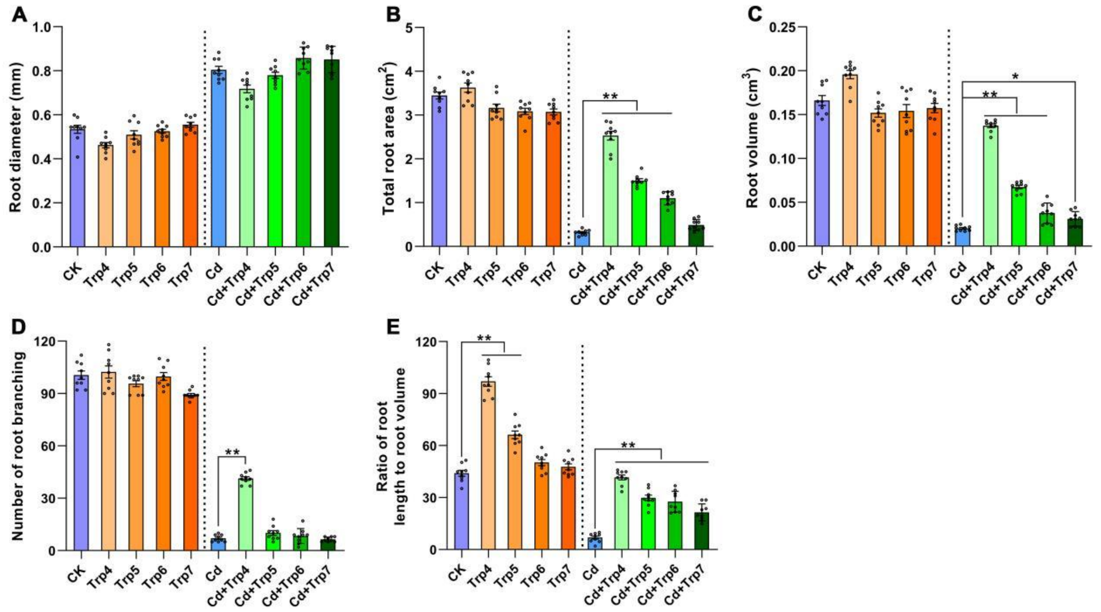

Figure S6. Root morphological traits of potato plantlets exposed to Cd stress supplemented with four low concentrations of melatonin (MT). (A–E) Root diameter, total root area, total root volume, root branching number, and the ratio of root length to root volume. Comparisons between selected pairs of treatments were conducted using Student's t-test. ** denotes extremely significant difference at $P < 0.01$. Error bars represent standard error, and scattered dots correspond to raw data of individual biological replicates.

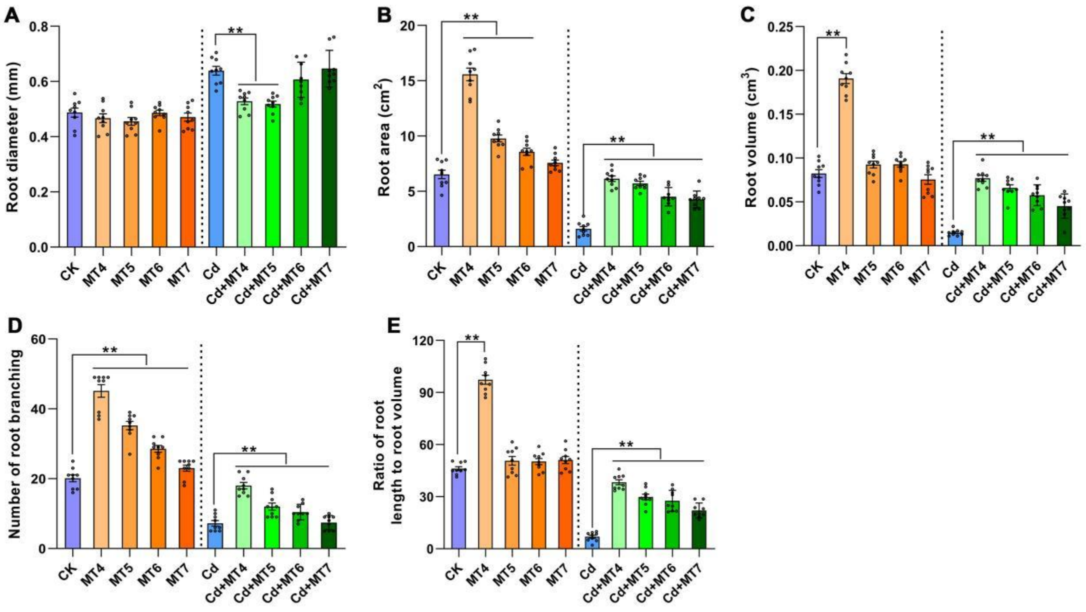

Figure S7. Root morphological traits of potato plantlets exposed to Cd stress supplemented with four low concentrations of tryptophan (Trp). (A–E) Root diameter, total root area, total root volume, root branching number, and the ratio of root length to root volume. Comparisons between selected pairs of treatments were conducted using Student's t-test. * denotes significant difference at $P < 0.05$; ** denotes extremely significant difference at $P < 0.01$. Error bars represent standard error, and scattered dots correspond to raw data of individual biological replicates.

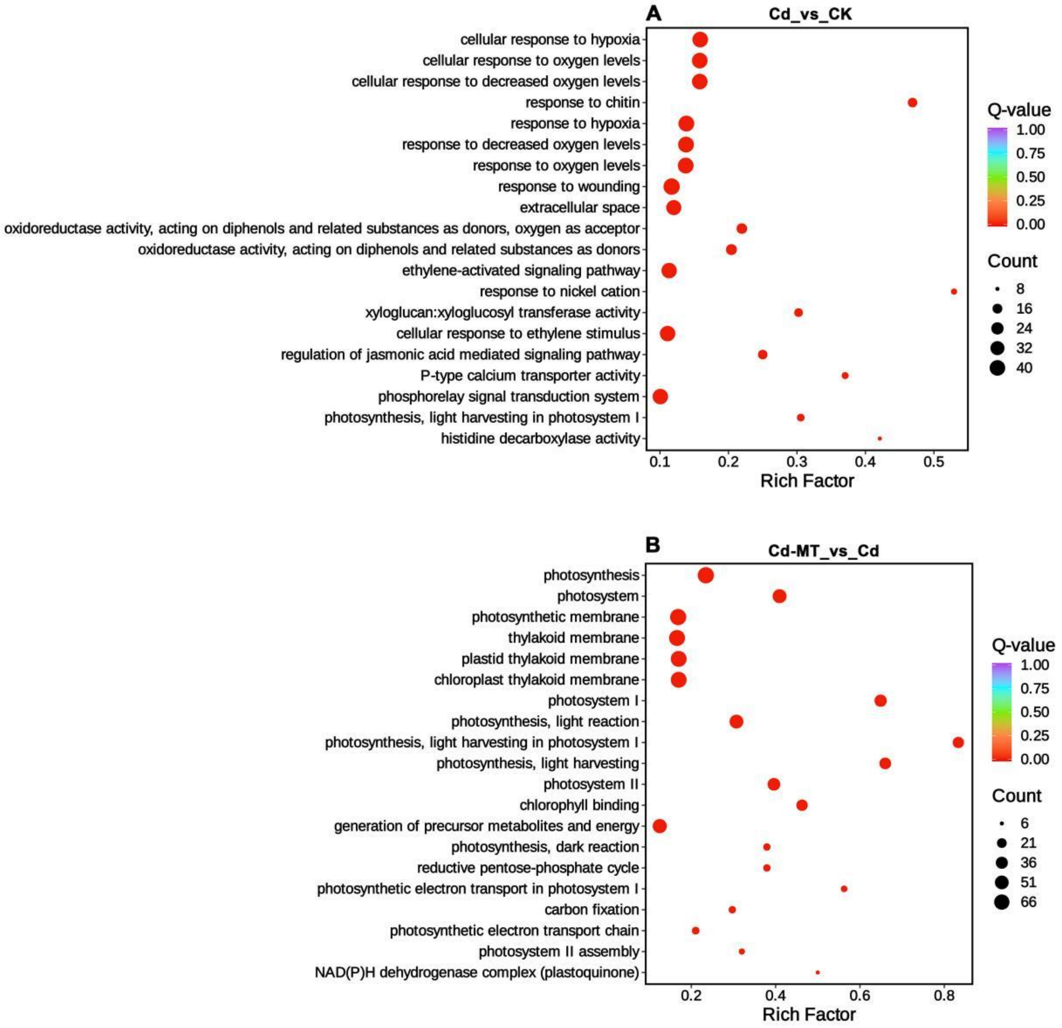

Figure S8. GO enrichment bubble analysis of differentially expressed genes (DEGs) in two comparison groups. (A) GO enrichment bubble plot of DEGs in Cd vs CK group. Bubble size represents the number of enriched genes, and color gradient corresponds to Q-value. (B) GO enrichment bubble plot of DEGs in Cd-MT vs Cd group.

Table S1. Sequences of primers in RT-qPCR.

Description	**Forward (5–3′)**	**Reverse (5–3′)**
StActin	GACAACGGAACTGCACGATC	TACGCTGAGCTTCATCACCA
StSAM-2	GATGGTGCTGATGAGGTGGA	TGCTGTCATCGGTGATGTTC
StCAS1	ACCTGGTGAGATTGATGTG	TCAGTTGCCCTTGGGCTGGT
StSWEET10	TGCAGAAGGTGAAGGAGCTG	TCCTTGGTGATGGTCCCTTT
StCYP81K2	TGTGGCTGGGTGTTGATGTTC	AGCAGATGAGGTTCATCAGG
StSDD1	GGTCGTGCTGCTGGTGTGATC	GCTTCCCCTTGCATCTCAGAC
StHMG1	ATGGCTGGTGTGGTGTGAA	TGCTGCTCTTGTGGTGATGT
StANNAT1	CCGTGTGAACAATAGTGCAA	CTCATGGCCTTCCTATGTTG
StNRT1.1	GCAATATGGTTCGTGAGGAG	TCCCTTCATGGTCTTGGTCA
